# Engineering protein processing of the mammary gland to produce abundant hemophilia B therapy in milk

**DOI:** 10.1038/srep14176

**Published:** 2015-09-21

**Authors:** Jianguo Zhao, Weijie Xu, Jason W. Ross, Eric M. Walters, Stephen P. Butler, Jeff J. Whyte, Lindsey Kelso, Mostafa Fatemi, Nicholas C. Vanderslice, Keith Giroux, Lee D. Spate, Melissa S. Samuel, Cliff N. Murphy, Kevin D. Wells, Nick C. Masiello, Randall S. Prather, William H. Velander

**Affiliations:** 1 National Swine Resource and Research Center & Division of Animal Science, University of Missouri, Columbia, MO 65211, USA; 2 Protein Purification and Characterization Laboratories, Department of Chemical and Biomolecular Engineering, 207 Othmer Hall, University of Nebraska, Lincoln 68588, USA; 3 State Key Laboratory of Reproductive Biology, Institute of Zoology, Chinese Academy of Sciences, Beijing, China, 100101; 4 Department of Animal Science, Iowa State University, Ames, IA 50011, USA; 5 ProGenetics, LLC, Blacksburg, VA, 24060, USA; 6 LFB USA, Inc.175 Crossing Blvd. Framingham, MA 01702, USA

## Abstract

Both the low animal cell density of bioreactors and their ability to post-translationally process recombinant factor IX (rFIX) limit hemophilia B therapy to <20% of the world’s population. We used transgenic pigs to make rFIX in milk at about 3,000-fold higher output than provided by industrial bioreactors. However, this resulted in incomplete γ-carboxylation and propeptide cleavage where both processes are transmembrane mediated. We then bioengineered the co-expression of truncated, soluble human furin (rFurin) with pro-rFIX at a favorable enzyme to substrate ratio. This resulted in the complete conversion of pro-rFIX to rFIX while yielding a normal lactation. Importantly, these high levels of propeptide processing by soluble rFurin did not preempt γ-carboxylation in the ER and therefore was compartmentalized to the Trans-Golgi Network (TGN) and also to milk. The Golgi specific engineering demonstrated here segues the ER targeted enhancement of γ-carboxylation needed to biomanufacture coagulation proteins like rFIX using transgenic livestock.

Hemophilia B is an X-linked, congenital bleeding disorder that is caused by a deficiency in the plasma protein coagulation factor IX (FIX). FIX deficiency occurs similarly in all races at about 1 in 25,000–35,000 males^1^. In 2012, it was estimated that only 28,000 of the world’s 140,000 hemophilia B patients were both diagnosed and properly treated by intravenous FIX replacement therapy^2^,^3^. Because 70–80% of hemophilia B patients reside in developing countries, they receive little or no treatment due to the high costs of recombinant and plasma-derived FIX. Although gene therapy may in the future reduce the need for FIX replacement therapy of FIX, both cost and the presence of pre-existing neutralizing antibodies could limit the universal application of this treatment option^4^.

FIX is a complex glycoprotein that circulates in plasma at trace levels of 5 μg/mL and is a member of the vitamin K-dependent (VKD) coagulation protein family^5^,^6^. The biosynthesis of FIX by hepatocytes begins with a precursor polypeptide that undergoes a number of post-translational modifications (PTMs). Most significant is the sequential VKD γ-carboxylation of pro-FIX and subsequent proteolytic cleavage of the propeptide^7^. In order to carboxylate the first 12 glutamic acids of the amino-terminus, the ER-transmembrane bound carboxylase must complex with the nascent pro-FIX via the propeptide^7^,^8^,^9^. The γ-carboxylation system requires redox cycling of the cofactor vitamin K using vitamin K epoxide reductase (VKOR)^10^,^11^. After transportation of the carboxylated pro-FIX into the TGN, the propeptide is removed by the paired basic amino acid cleaving enzyme (PACE) called Furin^12^. The presence of the propeptide or inadequate γ-carboxylation at the majority of residues within the amino terminus will render the secreted FIX non-functional^13^. Importantly, the autocatalytic maturation process for endogenous furin purposely compartmentalizes PACE activity to the TGN and prevents premature cleavage of pro-FIX in ER that would interrupt carboxylation^14^.

There are both cell and species specific limitations in the processing of PTMs needed for VKD protein functionality. This is illustrated (Supplementary Table S1) in a comparison of Baby Hamster Kidney (BHK)^15^ with pig mammary epithelial (PME) cells^16^,^17^,^18^. For example, when rFIX is made at the low rate of about 0.13 pg/cell/hour by PME cells, most of the rFIX is observed to be functional^17^. At the higher overall biosynthetic rates of 0.67 pg/cell/hour in BHK cells and 1.3 pg/cell/hour in PME cells, both cells reach a ceiling in the production rate of functional rFIX of about 0.13 pg/cell/hour^15^,^17^. With respect to species specific nature of this limitation in PME cells, much lower levels of functional rFIX and other VKD proteins have been made by mice and ruminant dairy livestock such as sheep^19^,^20^. Even at equivalent per cell capacity to make VKD proteins, the 2 to 3 orders of magnitude higher cell density of the mammary gland is a distinct advantage in secretory productivity over cultured animal cells^16^,^21^. This is seen in the bioreactor level output of functional rFIX by cultured BHK cells at about 0.12 μg/mL/hour while pig milk yields about 200 μg/mL/hour^17^.

The improvement of PTM in cultured animal cells has been demonstrated. In the case of BHK cells, γ-carboxylation of rFIX was improved 2.8-fold with the coexpression of recombinant VKOR (rVKOR)^15^. At the tissue level, previous studies by Drews *et al.* used mammary specific expression of rFurin in mice using a long murine Whey Acidic Protein (WAP) promoter to increase the proteolytic processing of internal dipeptides of the recombinant human protein C^22^. To our knowledge, the tissue specific engineering of PTM in livestock has not been reported. Here we present the complete processing of pro-rFIX to rFIX at high levels using the Golgi-targeted, coexpression of a soluble recombinant human furin (rFurin) with PACE specific activity in the pig mammary gland.

## Results

### Generating bigenic pigs with FIX and FURIN transgenes

**Figure 1:**
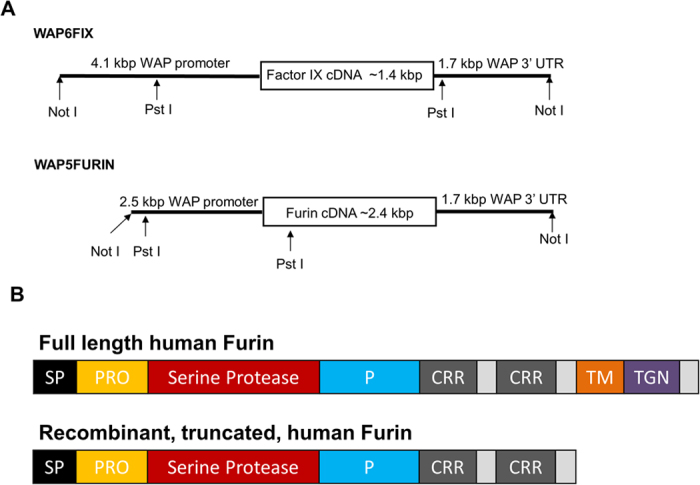
(**A**) Transgene construct schematic for long WAP-FIX (WAP6FIX) and short WAP-FURIN (WAP5FURIN). The cDNA for human FIX and truncated human furin cDNA were placed under control of the 4.1 (long) and 2.5 kbp (short) mammary specific Whey Acidic Protein (WAP) promoters, respectively. (**B**) Domain schematic of the primary structure of both native and truncated human furin. Signal Peptide (SP), Propeptide (PRO), Subtilisin-like Serine Protease domain, activation maturation domain (P), Cystine-rich region (CRR), trans-membrane domain (TM) and Trans Golgi Network Signal domain (TGN).

**Figure 2:**
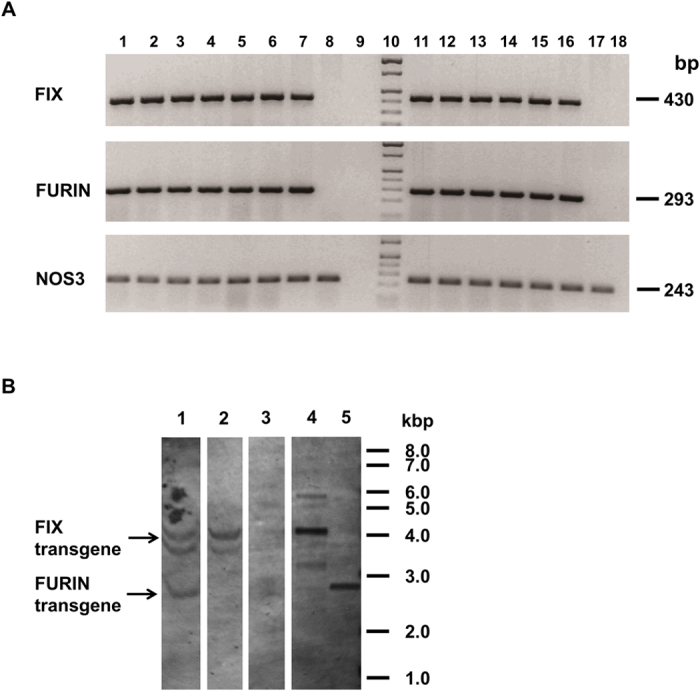
Panel (**A**) Genotyping of FIX and FURIN in the piglets of litter 78 and 79 by PCR. Eleven piglets from the two litters were bigenic for both transgenes; NOS3 was used as an endogenous gene positive control. Lane 1–6, 6 piglets of litter 78; Lane 7, positive control; Lane 8, wild type genomic DNA; Lane 9, negative control; Lane 10, DNA reference ladder; Lane 11–15, 5 piglets of litter 79; Lane 16, positive control; Lane 17, wild type DNA; Lane 18, negative control. The full images are shown as Supplementary Fig. S1A–C. Panel (**B**) Southern analysis of transgenic pig DNA using a WAP specific probe. 4.2 kbp fragment was WAP6FIX construct and 2.7 kbp fragment indicating the WAP5FURIN construct. Lane 1, Bigenic pig R185; Lane 2, monogenic pig K108; Lane 3, non-transgenic pig; Lane 4, FIX transgene standard applied at 5 copies; Lane 5, FURIN transgene standard applied at 5 copies; The arrows indicate FIX and FURIN transgenes, respectively. The original image is shown as Supplementary Fig. S1D.

Somatic-cell nuclear transfer (SCNT)^23^ was used to bioengineer both FURIN and FIX transgenes (Fig. 1A) into pigs. Three pregnancies resulted from embryo transfers to 6 surrogate recipients which produced a total of 15 founder male transgenic pigs (Supplementary Table S2A). All of these founder (F0) males were “bigenic” for both long WAP-FIX^18^ and short WAP-FURIN transgenes. The F0 male transgenic lines, Line A (78-2, 78-4), Line B (79-2) and Line C (80-4) were expanded by mating them with wild type Landrace sows to produce F1 offspring. These matings produced 26 of 64 offspring (41%) from 5 litters that were identified by PCR as bigenic F1 animals (Table 1 and Fig. 2A). Four female F1 animals were selected for milk production. In addition, the gene transformation and SCNT technique used here was capable of producing low copy number animals. For example, a Southern Blot Analysis of the DNA obtained from the tail of F1 bigenic female R185 (Fig. 2B) showed 2–3 copies of the FIX transgene and 2–3 copies of the furin transgene. When outbred with a nontransgenic boar, R185 produced two healthy litters with a total of 8 bigenic and 7 nontransgenic F2 offspring for a 57% transmission frequency (Supplementary Table S2B).

**Figure 3:**
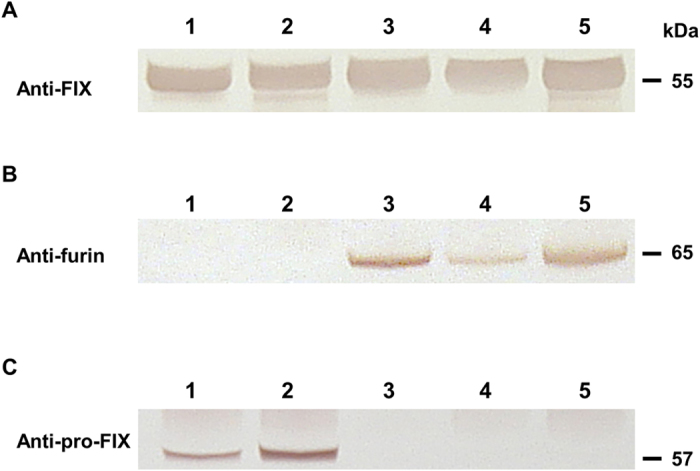
Western blot analysis of milk samples from monogenic and bigenic pigs. Detection of (**A**) rFIX antigen using anti-human FIX antibody; (**B**) rFurin antigen using anti-human furin antibody; (**C**) Pro-rFIX antigen using anti-human pro-FIX antibody. Milk sample pools from lactation days 5–35 of monogenic pigs (K75 and K101) and bigenic pigs (R175, R180 and R1014) were diluted with 200 mM EDTA 1: 1 and clarified as described in methods. Diluted milk sample pools: Lane 1, from pig K75; Lane 2, from pig K101; Lane 3, from pig R175; Lane 4, from pig R180; and lane 5, from pig R1014. 2 μL of each diluted milk sample pool was loaded. The full images are shown as Supplementary Fig. S3.

We compared the overall rFIX protein concentration in milk samples collected from the bigenic pigs to that of transgenic pigs containing only the long WAP-FIX gene^18^ (termed “monogenic”). Both the rFIX and endogenous protein content of the milks of the mono- and bigenic pigs throughout lactation were very similar (Fig. 3A, Table 2 and Supplementary Fig. S2, S3A). The endogenous protein composition of the milks of these transgenic pigs was similar to that reported for nontransgenic pigs^24^.

**Figure 4:**
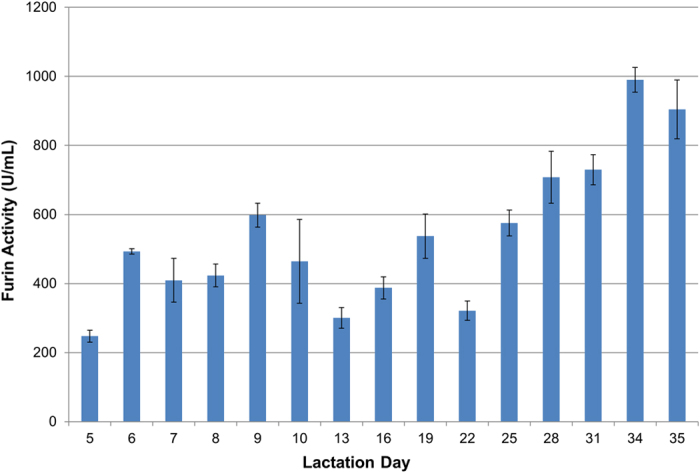
Furin activity in daily milk samples of bigenic pig R180. The furin activity in milk samples from every other lactation day of bigenic pig R180 was measured. One Unit of furin is defined as the cleavage of fluorogenic substrate, pERTKR-AMC, and producing 1 pmol AMC/min at room temperature. The error bars indicate the standard deviation (n = 3).

By using the short WAP promoter^25^,^26^ in the Furin transgene construction, we achieved a lower expression of the rFurin relative to rFIX that was more consistent with an enzyme to substrate ratio needed for PTM. The rFurin levels were estimated to be 12.5–50 mg/L with PACE activities ranging from 500 to 2000 U/mL (Table 2). In contrast, no furin protein or enzymatic activity was observed in any of the milks collected from monogenic pigs (Fig. 3B and Table 2). The rFIX to soluble rFurin ratio was estimated to be about 50–100 fold in all bigenic milks throughout lactation (Fig. 4, Table 2 and Supplementary Fig. S4).

### Pro-rFIX processing in monogenic and bigenic pigs

Western analysis indicated that about 9% to 27% of the total rFIX in the milk of monogenic pigs was pro-rFIX (Fig. 3C, Table 2). In contrast, no pro-rFIX was observed in bigenic milks (Fig. 3C). The pro-rFIX was immunopurified from the whole rFIX population that had been captured from monogenic milk samples using heparin affinity chromatography (Supplementary Fig. S5). The identity of the pro-rFIX was confirmed by both N-terminal amino acid sequence (Supplementary Table S3) and by its relative molecular weight that was 2 kDa more than the rFIX zymogen (Supplementary Fig. S5).

**Figure 5:**
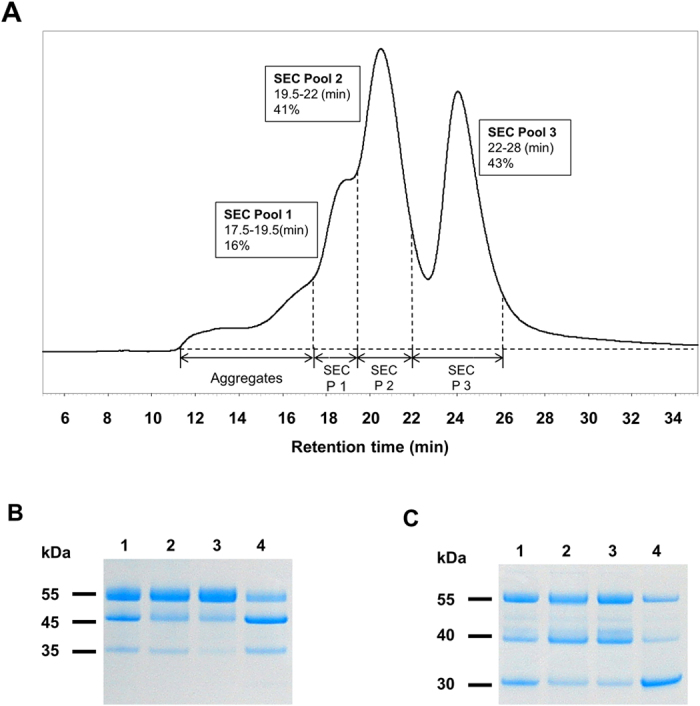
Separation of functional rFIX from non-functional and degraded rFIX using HPLC-SEC. Panel (**A**) HPLC-SEC elution profile of high acidic rFIX fraction from anion-exchange chromatography where fractions of each peak were collected as a pool. The rFIX mass percentage of each pool is shown as inset label. Panel (**B**,**C**) Non-reduced and reduced Colloidal blue stained SDS-PAGE. Lane 1, high acidic rFIX eluate from anion-exchange chromatography; Lane 2, SEC pool 1; Lane 3, SEC pool 2; and Lane 4, SEC pool 3, 2 μg of each sample was loaded.

The gamma-carboxylglutamic acid (Gla) content^5^,^27^,^28^ of rFIX and other VKD coagulation proteins generate compaction in holoprotein structure needed for biological activity^29^. Thus, we used high pressure size exclusion chromatography (HPLC-SEC) to isolate functional rFIX from differentially carboxylated subpopulations that were nonfunctional. This technique also separated highly carboxylated, but degraded rFIX that resulted from milk borne proteolysis (Fig. 5). The highly acidic but proteolyzed rFIX fraction isolated from all milks by HPLC-SEC was similar in mass percentage to intact and functional rFIX, and indicative of plasmin-like degradation^30^.

Our non-immunoaffinity purification methods could not separate highly carboxylated pro-rFIX from the functional rFIX zymogen that occurred in monogenic milks (Supplementary Fig. S6). The specific activity of the highly carboxylated, intact rFIX fractions made from monogenic milk were depressed to about 100 IU/mg due to a 20–30% content of pro-rFIX (Table 3). In contrast, the specific activity of the functional rFIX purified from the bigenic milk was 150–250 IU/mg and comparable to therapeutic grade, plasma-derived and recombinant FIX products^28^,^31^.

### *In vitro* characterization of soluble rFurin activity

**Figure 6:**
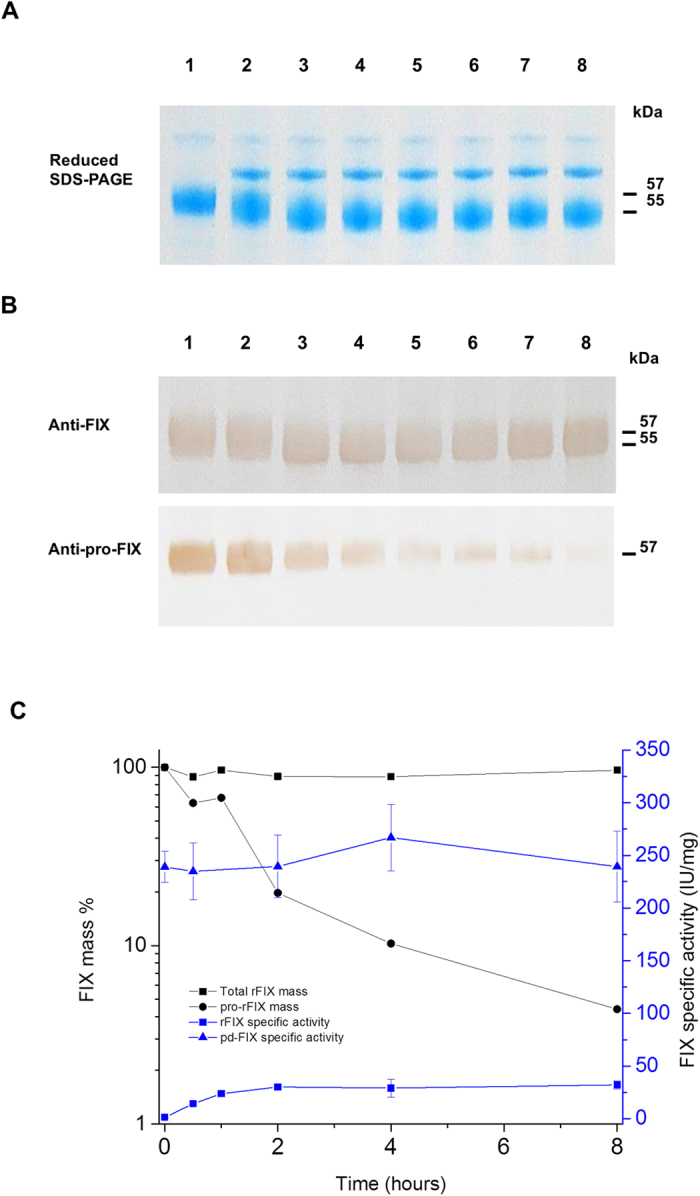
Time course study of *in vitro* pro-rFIX to rFIX processing by partially purified rFurin. (**A**) Reduced Colloidal blue stained SDS-PAGE. (**B**) Non-reduced western blots analysis detection of rFIX and pro-rFIX antigen using anti-human FIX and pro-FIX antibody respectively. Lane 1, immunopurified pro-rFIX (57 kDa), 1 μg; Lane 2–8, samples of a mixture of partially purified rFurin (0.075 U) and immunopurified pro-rFIX (1 μg) at reaction times = 0, 30 min, 1, 2, 4, 8 and 24 hr, respectively; The full images are shown as in Supplementary Fig. S7. (**C**) Time course plot of mass percent of various FIX species (left hand axis) and respective FIX specific activity (right hand axis). Solid black squares are total rFIX mass %; solid black circles are pro-rFIX mass%. The solid blue squares and triangles are time course FIX specific activity (IU/mg). The error bars indicate the standard deviation (n = 3); the values of mass and activity are shown as in Supplementary Table S6.

The PACE activity of the soluble rFurin was stable in whole milk or in the presence of both calcium ions and detergent (Supplementary Table S4). The need for detergent to stabilize the PACE specific activity of soluble rFurin (Fig. 1B) is consistent with the overall transmembrane associated nature of its function. The specificity of the PACE activity was further evidenced by the lack of proteolytic degradation or activation of intact rFIX or pd-FIX occurring when incubated with the rFurin (Fig. 6 and Supplementary Fig. S7).

We investigated the nature of the rFurin processing using *in vitro* treatment of the pro-rFIX by rFurin that was partially purified from the milk samples of the bigenic pigs (Supplementary Fig. S8). About 40% of the initial PACE activity observed in milk was recovered after four purification steps (Supplementary Table S5). The purified rFurin (Supplementary Fig. S4) exhibited a molecular weight of about 65 kDa and possessed an N-terminal amino acid sequence consistent with mature human furin (Supplementary Table S3). A high specific PACE activity, about 4 × 10^4^ U/mg, was measured for this rFurin product by amidolytic assay and this was consistent with the efficient conversion of immunopurified pro-rFIX *in vitro* by rFurin (Fig. 6).

After complete propeptide processing *in vitro* by partially purified rFurin, the specific activity of pro-rFIX samples immunopurified from the whole rFIX population in monogenic milk rose from none to about 35 IU/mg, (Fig. 6C and Supplementary Table S6). By assuming a specific activity of 200 IU/mg as seen with purified, functional rFIX from bigenic milk, we estimate that about 20% of the immunopurified pro-rFIX was functionally carboxylated. In addition, we added rFIX depleted milk from bigenic pig to monogenic pig milk as an additional pathway to convert pro-rFIX to rFIX zymogen before purification (Supplementary Fig. S9). This approach rapidly resulted in complete processing of pro-rFIX to a rFIX product having a specific activity that was more comparable to that purified from bigenic milk (Table 3). The overall yield of functional rFIX purified from monogenic milk treated by rFIX-stripped-bigenic milk was similar to those obtained from milk samples of monogenic and bigenic pigs alone. This indicated that γ-carboxylation was not significantly interrupted by the high levels of expressed rFurin.

Using the kinetics obtained by our *in vitro* studies of the rFurin, we estimate that complete propeptide processing of the pro-rFIX would occur within 30 minutes or less residing at 38.7–40 °C temperature of the mammary gland of the bigenic pigs. This is consistent with the complete processing of pro-rFIX to rFIX observed in all bigenic milk as typical pig milk letdown occurs about every hour (Table 2 and Fig. 3).

## Discussion

These studies demonstrate that the pig mammary gland can be bioengineered to greatly improve PTM efficiency for a complex recombinant protein made at high levels in milk. We chose two different WAP promoters so as to express rFIX and rFurin at a desirable substrate to enzyme ratio while taking advantage of the mammary specific regulation previously known for these WAP formats^17^,^25^,^26^. This resulted in F1 female pigs making completely processed pro-rFIX while displaying a generally healthy phenotype. This healthy phenotype was evidenced by: normal pregnancies and litters; sustained, extended lactations; and milks with normal endogenous protein composition (Supplementary Fig. S2 and Table S2B, S7). This contrasted the milk composition previously observed in transgenic pigs expressing human protein C which were accompanied by elevated levels of IgG, IgM, IgA and transferrin^24^. With respect to the mammary specific expression of the WAP-FIX or Furin transgenes, only very low levels of ectopic expression have been previously reported for WAP promoter based constructs^24^,^26^. This low but detectable ectopic expression observed in mice occurred with no apparent adverse effects^24^. Thus, due to the healthy phenotype observed here in all animals, we chose not to evaluate the ectopic expression of either the WAP-FIX or Furin cDNA transgenes.

The reliability of using the mammary gland of a transgenic livestock for producing biotherapeutics is centered upon the combination of stable genotype, healthy phenotype and recombinant protein expression^32^. The DNA transfection and SCNT techniques used here to make transgenic pigs were amenable to making lower copy numbers animals which typically result in long term transgene stability. As an example, the R185 F1 animal had a low copy transgene number for both transgenes exhibited a Mendelian transmission frequency of the transgenes collectively from two F2 litters while producing high levels of rFIX and rFurin and fully processed pro-rFIX. With respect to the future goal of obtaining an Investigational New Drug application to USFDA for manufacture of rFIX^32^, the use of the F1 pigs or their source somatic cell lineage would require further study such as the determination of the sequence of the transmitted transgenes.

The potential to harness the extraordinary secretory capacity of livestock making complex proteins, like rFIX, hinges on its ability to properly make PTMs. In the case of rFIX, the basal capacity of the pig to make the sequential PTMs of γ-carboxylation and propeptide cleavage is generally better than in ruminant dairy livestock^20^. However, at a milk production of only 200 liters per year, the pig mammary gland must more efficiently make these PTMs if is to be used for biopharmaceutical production. We used an improved mammary specific promoter to elevate the levels of rFIX biosynthesis ten-fold over that where fully functional rFIX was obtained in milk (Supplementary Table S1)^18^. While the lower secretory rate of about ≤200 μg/mL/hr yielded complete γ-carboxylation and propeptide cleavage, these processes became rate limited at about >1000 μg/mL/hr^17^. In all animals studied here, the level of propeptide cleavage processing resulting from soluble rFurin expression was well beyond that needed for biopharmaceutical production while not preempting γ-carboxylation in the ER (Fig. 3 and Tables 2 and 3). Important to this goal, was the simultaneous biosynthesis of rFurin and rFIX protein at an appropriate PACE enzyme to substrate ratio that was sustained throughout lactation. This eliminated the need for an additional chromatography step to remove pro-rFIX.

About 10–20% of total 10–30% pro-rFIX found in monogenic milk was sufficiently carboxylated to support rFIX functionality. Based upon *in vitro* processing of immunopurified pro-rFIX, the additional processing of the pro-rFIX was estimated to represent an additional 0.4–1.2% functional rFIX to the overall yield. This predicted gain in functional rFIX was not discernable when comparing bigenic, monogenic, and monogenic milks treated with rFIX stripped bigenic milks as all resulted in 2–3% overall yield (Table 3). The full benefit of converting pro-rFIX to functional rFIX by tissue engineering of rFurin will be gained after the level of γ-carboxylation is raised along with better management of proteolytic degradation. Plasmin is the major protease of milk^33^ and the proteolytic products we observed were indicative of plasmin proteolysis^30^ (Fig. 5). We estimate that the overall yield of functional rFIX can be potentially doubled if proteolytic degradation can be better managed within the gland and during purification by addition of serine protease inhibitors and adjustment of pH.

Past studies of the expression of rFIX in mammalian cells in culture have demonstrated that the chief limitation in γ-carboxylation resides in the redox shuttle system that is mediated by the ER transmembrane protein VKOR^10^,^15^. It is noted that endogenous pro-Furin is autoactivated and fully matured in the TGN to prevent PACE activity form occurring in the ER where γ-carboxylation is performed^14^. This naturally prevents premature processing of the propeptide before γ-carboxylation is completed. In contrast, the truncated rFurin was engineered without a transmembrane domain (TM) and TGN signal domain (Fig. 1B) therefore making it soluble in both ER and TGN compartments. However, in spite of the high expression level of rFurin, no significant impact on both overall rFIX expression level and basal levels of γ-carboxylation occurred. This was evidenced by the similar yields of functional rFIX obtained from bigenic, monogenic, and monogenic milks treated with rFurin *in vitro* (Table 3).

**Figure 7:**
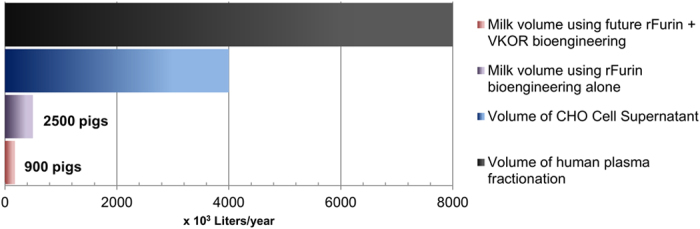
Estimated annual FIX source volumes needed to meet the worldwide clinical needs for hemophilia B patients in developing countries (estimated 4 billion IU FIX required using prophylactic therapy). The estimated concentration of FIX in each source: plasma (1 IU mL^−1^); CHO cell bioreactor (2 IU mL^−1^); milk of transgenic pigs engineered with rFurin alone (40 IU mL^−1^); future milk of transgenic pigs engineered with both rVKOR and rFurin alone (105 IU mL^−1^) . An overall purification yield of 50% is assumed for plasma and cell culture while degradation of rFIX by milk born proteases reduces the overall yield to about 20%. About 2,500 pigs bioengineered with rFurin and 900 pigs bionengineered with rVKOR and rFurin is the estimated amount of pigs need to produce sufficient milk to provide prophylactic therapy for hemophilia B patients in developing countries worldwide.

The expression of rVKOR in BHK cells^15^ has improved the functional rFIX levels by about 2.8-fold and we estimate that expressing both rVKOR and rFurin could increase functional rFIX levels to about 500–600 μg/mL/hour (Supplementary Table S8). While the native capacity of the pig mammary gland to make 20 kg rFIX/year would require 2500 milk pigs annually to satisfy clinical needs of 80% of the Hemophilia B population worldwide, only about 900 milking pigs would be needed after bioengineering with rVKOR and rFurin (Fig. 7 and Supplementary Table S8). A chief barrier to the production of biopharmaceuticals is the capital investment in manufacturing facilities which typically costs about USD $0.6 billion per product and 5 years to build^34^. It is estimated that the capital investment for transgenic livestock biopharmaceutical production is <10% of that needed for cell culture production facilities at an equivalent capacity^35^,^36^,^37^. The recent approval of recombinant human antithrombin III made in the milk of transgenic goats^38^,^39^ by the US FDA and European Medicines Evaluation Agency (EMEA) bodes well for the future implementation of biopharmaceutical production using transgenic livestock.

## Methods

All buffer components were purchased from VWR International LLC (Radnor, PA) or Thermo Fisher scientific (Waltham, MA) or Sigma (St. Louis, MO) or Invitrogen (Life technologies, Grand Island, NY) unless otherwise stated. All of the following solutions and media used in fetal fibroblast collection, transgenic cells producing, oocyte maturation, SCNT, and embryo reconstruction were filtered by using a 0.22 μm filter. All of following buffers and samples loaded on the high pressure liquid chromatography (HPLC) were filtered by using 0.45 μm filter. All animal procedures were performed with an approved University of Missouri Animal Care and Use (ACUC) protocol. All animal experiments were carried out in accordance with the Guide to the Care and Use of Laboratory Animals, and under a protocol approved by the University of Missouri Institutional Animal Care and Use Committee. Unless stated otherwise, the following purifications were performed on a BioCAD Vision chromatography station (Applied Biosystems, Grand Island, NY). In order to minimize degradation, purification processes were performed at 4 °C. The pd-FIX (Mononine, CSL Behring, King of Prussia, PA) and CHO-rFIX (BeneF9, Pfizer, New York City, NY) used in the study were expired for clinical use; however, when used in experiments, exhibited full pro-coagulant activity. Both were kind gifts from James Brown (Lincoln, NE).

### Fetal Fibroblast Collection

Landrace fetal fibroblasts cells (FFCs) were collected as described^23^ with some modifications. Briefly, after removing the head, intestine, liver, limbs, and heart, the fetus was minced and digested individually in 20 mL of digestion media (Dulbecco’s Modified Eagle Medium (DMEM) supplemented with 15% (v/v) FBS, 200 units/mL collagenase and 25 Kunitz/mL DNaseI) for 5 hrs at 38.5 °C and 5% CO_2_ in air. Digested cells were washed with DMEM, 15% fetal bovine serum (FBS) (Lot number: ASM31113, Catalog: SH30071.03, Hyclone, Logan, UT) with 10 μg/mL gentamicin, cultured overnight, then collected and frozen at −80 °C in aliquots in FBS with 10% dimethyl sulfoxide (DMSO) and stored in liquid nitrogen.

### Production of gene constructs

The production of the Long WAP-FIX (WAP6FIX) was previously detailed^18^,^40^. The production of the Short WAP-FURIN (WAP5FURIN) transgenes made here used the parent Long WAP-FURIN construct^22^ and short WAP-FIX construct^17^ as templates. A 2.5 kbp *Eco RI-Kpn I* WAP promoter element^17^ was installed at the *Kpn I* site of the 2.47 kbp furin cDNA that has been previously described^22^. WAP-FIX and WAP-FURIN constructs were linearized by *Not I*. After enzyme digestion, constructs were gel purified and stored at −20 °C until transfection. Both Long WAP-FIX and short WAP-FURIN transgene constructions are shown in Fig. 1A.

### Production of transgenic cells

Early passage number Landrace FFCs (1–2) were cultured in cell culture medium (DMEM supplemented with 15% (v/v) FBS, 2.5 ng/mL basic fibroblast growth factor (Sigma) and 10 μg/mL gentamicin) overnight and grown to 75–85% confluency. Media was replaced 4 hours prior to transfection. FFCs were washed for 1–2 min with phosphate buffered saline (Life Technologies) and harvested with 0.05% trypsin-EDTA (Life Technologies; 1 mL per 75 cm^2^ flask). Cells were resuspended in cell culture medium, pelleted at 600 × g for 10 min, resuspended in 10 mL Opti-MEM (Life technologies), and then quantified by using a hemocytometer and repelleted. Cells were resuspended in transfection media (75% cytosalts [120 mM KCl, 0.15 mM CaCl_2_, 10 mM K_2_HPO_4_; pH 7.6, 5 mM MgCl_2_]^41^ and 25% Opti-MEM (Gibco BRL Grand Island, NY)) and quantified again and the cell concentration was adjusted to 1 × 10^6^ cells/mL. Two hundred microliters cells were co-transfected by electroporation with linearized WAP-FIX (2 μg), WAP-FURIN (2 μg) constructs. Electroporation was conducted as previously optimized^42^. Briefly, each electroporation utilized three consecutive 250-V, 1-ms square wave pulses administered through a BTX ECM 2001 (BTX, San Diego, CA) in 2 mm gap cuvettes. After electroporation, cells were plated in 100 mm dishes at the concentration of 3,000 cells per dish in cell culture medium. After 36 hours, cells were selected by the addition of geneticin (G418; 400 μg/mL) for 10–14 days until the formation of cell colonies. Genomic DNA from the cell colonies was used to verify the presence of both genes by PCR. These cells then were stored in liquid nitrogen until used as donor cells for somatic cell nuclear transfer (SCNT).

### Oocyte maturation, SCNT, and embryo reconstruction

Fibroblast cell lines identified to have stable integration of both FIX and FURIN constructs were used as donor cells for SCNT into enucleated oocytes followed by electrical fusion and activation^23^. In brief, cumulus-oocyte cell complexes (COCs) were received in Phase I maturation medium from ART Inc. (Madison, WI) approximately 24 hours after harvest. COCs were then cultured in fresh Phase II maturation medium for 16 h. Maturation was in a humidified atmosphere of 5% CO_2_ at 38.5 °C. Expanded COCs were then vortexed in 0.1% hyaluronidase in Hepes-buffered Tyrode’s medium containing 0.01% polyvinyl pyrrolidone for 4 min to remove the cumulus cells. Only oocytes having a visible first polar body (PB) with uniform cytoplasm were selected and placed in fresh manipulation medium (25 mM Hepes-buffered TCM199 with 3 mg/mL bovine serum albumin (BSA)) containing 7.5 μg/mL cytochalasin B overlaid with warm mineral oil. SCNT was conducted^23^ by removing the PB, MII chromosomes and a small amount of surrounding cytoplasm of the oocyte by using a beveled glass pipette with an inner diameter of 17–20 μm. After enucleation, a donor cell was injected into the perivitelline space and placed adjacent to the recipient cytoplasm. The reconstructed embryos were fused and activated with 2 DC pulses (1 sec interval) of 1.2 kV/cm for 30 μsec provided by a BTX Electro-cell Manipulator 200 in fusion medium (0.3 M mannitol, 1.0 mM CaCl2, 0.1 mM MgCl_2_, and 0.5 mM Hepes, pH adjusted to 7.0–7.4). After fusion and activation, only the fused embryos were cultured in four well plates (Nunc, Denmark) containing 500 μL of PZM3 with 0.3% BSA and 500 nM Scriptaid at 38.5 °C and 5% CO_2_ in humidified air for 14 to 16 hours, until embryo transfer^43^.

### Embryo transfer and piglet production

More than 100 SCNT zygotes were surgically transferred to the oviducts of each surrogate on the day of, or one day after, the onset of estrus. Ultrasound was used to monitor the pregnancy. Piglets were delivered via cesarean section from surrogates by day 114–116 of gestation. Piglets are processed immediately and tissue samples were collected for establishment of cell lines and PCR genotyping. Piglets were then hand-raised until weaning (3–4 weeks of age). After confirming genotype and reaching sexual maturity, the founders with both FIX and FURIN were mated to wild type pigs for production of animals for milking and phenotyping.

### PCR genotyping assays

The integration of transgenes in the founders and offspring was conducted by using 10 ng of genomic DNA for each 25 μL PCR reaction (Mastercycler Pro; Eppendorf, Hauppauge, NY). GoTaq polymerase (Promega, Madison, WI) was used as recommended by the manufacturers. PCR primers (Supplementary Table S9) were used in under PCR cycling conditions of: 95 °C for 3 minutes followed by 30 cycles at 95 °C for 15 seconds, 58 °C for 30 seconds, and 72 °C for 45 seconds, 95 °C for 3 minutes followed by 30 cycles at 95 °C for 15 seconds, 59 °C for 30 seconds, and 72 °C for 45 seconds. 95 °C for 3 minutes followed by 30 cycles at 95 °C for 15 seconds, 59 °C for 30 seconds, and 72 °C for 45 seconds.

### Southern Blot

The biotinylated probe was produced by PCR using KPL (Rockville, MD) Dectector PCR Biotin Label kit and the following primers 5′-ggttgccaaggtctgggggc and 5′-ggctcgagtccgggctacat. The position of the probe was shown in Fig. 1A. To prepare the blot, DNA isolated from blood samples was digested with Pst I from NEB (Ipswich MA) overnight along with plasmid derived standards digested with Pst I as well. Samples were loaded on a 0.8% agarose gel along with biotinylated molecular weight markers NEB biotinylated 2 log DNA markers. After 3 h at 120 v, the DNA was transferred to a Magna Charge nylon membrane from Osmonics using a vacuum apparatus system. After transfer, the blot was UV crosslinked and hybridized at 42 °C overnight using KPL hybridization solution and manufacturer’s instructions. Blot was detected using a streptavidin linked alkaline phosphatase kit and manufacturer’s instructions.

### Pig milking

Transgenic pigs milk was collected as previously described^44^. Milk samples were collected twice daily on the following days (D5-10, then every three days until D35). Piglets were removed from the mother 30 minutes before milk sample collection and returned following sample collection. An injection of Oxytocin was given to the sow 10–15 minutes before sample collection. No inhibitors or EDTA were added after collection. The milk samples were stored in the freezer at −80 °C until analysis.

### Sodium Dodecylsulfate-Polyacrylamide Gel Electrophoresis (SDS-PAGE)

Samples were analyzed by using 12% Bis-Tris Novex acrylamide precast gels and the SureLock XL apparatus (Life Technologies). Briefly, samples were mixed with LDS sample buffer (4x) and with or without additional reducing agent (10x) (reduced condition) and deionized water followed by heating at 74 °C for 10 min. The Gels were run at 200 Volts for 1 hour at 4 °C. Then gels were stained with Colloidal Blue (Life Technologies).

### Western blot analysis

Gels were first electro-blotted onto poly vinylidene fluoride (PVDF) membranes using a Transblot SemiDry Transfer Cell (BioRad, Richmond, CA) for 30 min at 25 volts. Total rFIX (including pro-rFIX, rFIX zymogen and degraded rFIX) was probed by rabbit polyclonal antibody (Pab) anti-human FIX (Sigma) and rFurin was probed by using rabbit Pab anti-human furin (476–490) (Sigma). A mouse monoclonal antibody (Mab), PROFIX 3D2.6H12 anti-propeptide of human pro-FIX (anti-pro-FIX) (Green Mountain Antibody, Burlington, VT), was used to detect pro-rFIX. A secondary antibody (HRP-conjugated) anti- rabbit and mouse IgG (Sigma) was used to detect the primary antibody, respectively. Then, color was developed using chromogenic DAB substrate (Thermo, Rockford, IL) or Immun-Star HRP buffer and enhancer (Bio-Rad, Hercules, CA) with BioMax Light-1 Film (Kodak, Rochester, NY).

### Quantification of rFIX and pro-rFIX in transgenic milk

Western blots were performed as described before. Mononine or immunopurified pro-rFIX standard was loaded along with the transgenic milk samples (in triplicate) as calibrators. Images of the blots were analyzed by using Image J^45^.

### Processing pro-rFIX in milk samples of monogenic pig

Milk samples of bigenic pigs (contains soluble rFurin) was first incubated heparin sepharose fast flow (FF) (GE Healthcare Bio-Sciences AB, Sweden) resin at 1:1 (v/v) ratio for 30 min at 4 °C. The supernatant was collected (rFIX stripped) and was then mixed with monogenic milk sample (with certain amount pro-rFIX) at 1:4 (v/v) ratio for 2 hour at 4 °C. All above milk samples were not treated with EDTA or any protease inhibitors. Conversion of pro-rFIX to zymogen after processing was analyzed before purification.

### Purification of functional rFIX from transgenic milk samples

Functional rFIX was purified using procedures adapted from previous study^18^. In brief, clarified transgenic milk samples were loaded onto a heparin hyper D column, 260/200 (GE Healthcare Bio-Sciences AB, Sweden) mixed with 20 mM Tris, 50 mM NaCl, pH 7.4 at 1:3 (v/v) ratio. Whole rFIX population was eluted with 500 mM NaCl after pre-wash with 200 mM NaCl. Or, milk samples were loaded on immunoaffinity FIX select (camelid IgG anti-FIX) column 260/200 after mixed with the FIX select equilibrium buffer (20 mM imidazole, 150 mM NaCl, pH 7.0) at 1:3 (v/v) ratio. Whole rFIX population was then eluted with 2 M MgCl_2_. Fractions were then loaded on Q sepharose 260/200 (GE Healthcare Bio-Sciences AB, Sweden) with 20 mM imidazole pH 7.0, 0.1 M ammonium acetate. High acidic (certain carboxylated) rFIX was eluted at 800 mM ammonium acetate after pre-wash with 537 mM ammonium acetate. High acidic fractions were then loaded on a high pressure size exclusion (HPLC-SEC) column 7.8/300, TSK gel G3000SW_XL_, (particle size, 5 μm; Pore size, 250 Å) (Tosoh Bioscience, LLC) and run in 20 mM Tris base, 200 mM NaCl, 10 mM CaCl_2_, pH 7.0. Fractions of peak with normal FIX specific activity (150–250 IU/mg) were pooled as the functional rFIX.

### Purification of Pro-rFIX from monogenic milk

Whole rFIX population was purified from the monogenic milk (contains pro-rFIX) as described above. Then fractions were loaded on the antibody anti-propeptide of human pro-FIX (anti-pro-FIX) Green Mountain Antibody, Burlington, VT) immobilized Protein G Sepharose FF (GE Healthcare Bio-Sciences AB, Sweden) column 10/127. The pro-rFIX was eluted by using 0.1 M glycine, pH 2.8 and then neutralized with 1:10 (v/v) 1 M Tris, pH 8.5.

### Quantification of rFIX specific activity

FIX specific activity was determined using the activated partial thromboplastin time (aPTT) assay^18^. Briefly, 50 μL each of PTT Automate 5 reagent (Diagnostica Stago, Inc., Parsippany, NJ, USA), FIX deficient plasma (George King Bio-Medical, Overland Park, Kansas, USA), and a sample was added to a cuvette and incubated at 37 °C for 3 min. 50 μL of 25 mM CaCl_2_ was then added and the clotting time was measured using the STart Hemostasis Analyzer (Diagnostica Stago, Inc., Parsippany, NJ). Normal human plasma (Diagnostica Stago, Inc., Parsippany, NJ) was used as the standard, assigning 1 unit of FIX clotting activity per mL of plasma. The concentration of purified rFIX was determined by measuring the optical density at 280 nm in a 1 cm quartz cuvette and using an extinction coefficient, ε^1%^ = 13.4. Each reference and FIX sample was analyzed in triplicate.

### NH2-terminal sequencing of pro-rFIX, rFIX and rFurin

Amino acid sequencing was performed at the University of Nebraska Medical Center, Protein Structure Core Facility. Protein samples were subjected to SDS-PAGE and transferred to PVDF as described above. The membrane was stained with Colloidal Blue and bands excised and subjected to Edman degradation using Applied Biosystems Procise protein sequencer, a Hitachi 8800 Amino Acid Analyzer and a Michrom MAGIC HPLC equipped with a Diode Array Detector.

### Purification of rFurin from bigenic milk

Milk samples from bigenic pigs (R185 and/or R1014 lactation pool) was clarified by mixing with 20 mM HEPES, 50 mM NaCl, 2 mM CaCl_2_, 0.1% (w/v) Brij-35, pH 7.5, at 1:4 (v/v) ratio and then centrifuging at 12,000 rpm, 4 °C for 20 min. Clarified milk samples were loaded on the DEAE FF column 10/127 (GE Healthcare Bio-Sciences AB, Sweden) and rFurin fractions were eluted at 140 mM NaCl. Then fractions were loaded on a ceramic hydroxyapatite (CHT) column (Bio-Rad, Hercules, CA) 10/127 in 20 mM HEPES, 2 mM Na_2_HPO_4_, 0.1% (w/v) Brij-35, pH 7.5. and all rFurin was in flow through. The fractions were loaded on Q Sepharose FF column (GE Healthcare, Bio-Sciences AB, Sweden) 10/127 in 20 mM HEPES, 50 mM NaCl, 2 mM CaCl_2_, 0.1% (w/v) Brij-35, pH 7.2 and rFurin samples were finally eluted with 140 mM NaCl.

### Quantification of furin activity

Fluorometric assays were carried out essentially as described^46^. 7- amino, 4-methyl coumarin (AMC) concentration from 0.39 μM to 25 μM (calibrators) and test samples were in 20 mM HEPES, 2 mM CaCl_2_, 0.1% (w/v) Brij-35, pH 7.5. Fifty μL 100 μM substrate, L-PyroGlu-Arg-Thr-Lys-Arg-AMC (pERTKR-AMC) (R&D systems, Minneapolis, MN, USA) was incubated with the 50 μL test samples at room temperature. Fluorescence was measured by Cary Eclipse Fluorescence Spectrophotometer (Agilent Technologies, USA) at λex 342 nm and λem 440 nm with kinetic mode for 20 mins. 1 Unit of PACE/furin specific activity is equal to generation of 1 pmol AMC/min at room temperature. Each milk sample was applied in the assay in triplicate.

### Time course investigation of propeptide of pro-rFIX processing by rFurin

Fifteen units partially purified rFurin was added into 200 μg purified pro-rFIX sample in 20 mM HEPES, 2 mM CaCl_2_, 0.1% (w/v) Brij-35, pH 7.5. At each time point (0, 30 min, 1, 2, 4, 8 and 24 hr), the reaction was quenched by taking 20 μL sample (containing originally 1 μg pro-rFIX and 0.075 U rFurin) and mixed with LDS sample buffer (4x) with or without additional reduced agent (10x) for non-reduced, reduced SDS-PAGE respectively. An additional 50 μL sample was quenched by 50 μL 20 mM sodium citrate, pH 7.4 for FIX specific activity (aPTT) assay. The reaction was performed at room temperature (25 °C). Reaction samples at each time points were analyzed in triplicate.

## Additional Information

**How to cite this article**: Zhao, J. *et al.* Engineering protein processing of the mammary gland to produce abundant hemophilia B therapy in milk. *Sci. Rep.* **5**, 14176; doi: 10.1038/srep14176 (2015).

## Supplementary Material

Supplementary Information

## Figures and Tables

**Table 1:** Production of F1 bigenic WAP-FIX and WAP-Furin pigs from F0 males.

**F0 male**	**Litter number**	**Offspring**	**Bigenic**	**Nontransgenic**
78-2	122	16	6	10
78-4	123	15	1	14
79-2	125	10	4	6
80-4	23, 129	23	15	8
Total	5 Litters	64	26 (41%)	38 (59%)

**Table 2:** Total rFIX, pro-rFIX and rFurin in milk samples from monogenic and bigenic pigs*.

**Milk source**	**PIG ID**	Total rFIX (mg/mL)**	**Pro-rFIX (mg/mL)**	**Pro-rFIX %**†	**Furin Activity (U/mL)**††
Monogenic Pigs	K75	2.30 ± 0.05	0.21 ± 0.05	9%	Not Detected
	K96	1.63 ± 0.36	0.25 ± 0.04	15%	
	K101	1.50 ± 0.02	0.40 ± 0.10	27%	
Bigenic pigs	R175	2.23 ± 0.07	Not Detected		1402 ± 40
	R180	1.60 ± 0.35			539 ± 217
	R1014	2.22 ± 0.06			2214 ± 33

The concentration and activity values are mean ± SD (n = 3).

^*^Monogenic pigs contain only the WAP-FIX transgene; bigenic pigs contain both WAP-FIX and WAP-Furin transgenes. Pooled milk samples were taken from select days over the course of lactation days (up to 35 days).

^**^Total rFIX contains both rFIX zymogen and pro-rFIX.

^†^The percentage indicates the mass percentage of pro-rFIX in total rFIX.

^††^One unit of furin specific activity is defined as the cleavage of fluorogenic substrate pERTKR-AMC and producing 1 pmol AMC/min at room temperature.

**Table 3:** rFIX specific activity and overall purification yield for monogenic and bigenic pig milk.

**rFIX source**	**FIX Specific activity (IU/mg)**	**Yield of Functional rFIX %**
Monogenic milk*	107 ± 7	2.2**
Bigenic milk†	216 ± 14	2
rFurin treated monogenic milk††	173 ± 31	2.5

The specific activity values are mean ± SD (n = 3).

^*^Monogenic pig K108 milk lactation pool was used in the purification.

^**^The yield of functional rFIX after purification from monogenic milk of 2.2% was adjusted for 25% pro-rFIX content (Supplementary Fig. S6) where the total pro-rFIX plus rFIX gave an overall yield of 2.9%. As determined by a single stage clotting assay, the functional rFIX species contained in the monogenic milk product was estimated to have a specific activity of 143 ± 7 IU/mg where the pro-rFIX species contributed no activity.

^†^Bigenic pig R185 milk lactation pool was used in the purification.

^††^Pro-rFIX in the monogenic milk sample was converted to the rFIX zymogen before purification by treating the monogenic milk sample with rFIX stripped bigenic milk sample containing rFurin. Monogenic pig K82, K89, K92, K102 and K108 whole milk lactation pools were mixed with Bigenic pig R185 or R175 whole milk lactation pools at 4:1 (v/v) ratio before purification. No pro-rFIX was detected in the sample after this treatment.
