# Supplementary material for: Engineering protein processing of the mammary gland to produce abundant hemophilia B therapy in milk
Source: Sci Rep. 2015 Sep 21;5:14176. doi: 10.1038/srep14176 (PMC4585688; doi:10.1038/srep14176)
Supplement: Supplementary Information [file srep14176-s1.pdf]

Supplementary Information

Engineering protein processing of the mammary gland to produce abundant hemophilia B therapy in milk

Jianguo Zhao[1,3], Weijie Xu[2], Jason W. Ross[1,4], Eric M. Walters[1], Stephen P. Butler[5], Jeff J. Whyte[1], Lindsey Kelso[1], Mostafa Fatemi[2], Nicholas C. Vanderslice[2], Keith Giroux[1], Lee D. Spate[1], Melissa S. Samuel[1], Cliff N. Murphy[1], Kevin D. Wells[1], Nick Masiello[6], Randall S. Prather[1] & William H. Velander[2]

[1] National Swine Resource and Research Center & Division of Animal Science, University of Missouri, Columbia, MO 65211, USA

[2] Protein Purification and Characterization Laboratories, Department of Chemical and Biomolecular Engineering, 207 Othmer Hall, University of Nebraska, Lincoln 68588, USA

[3] State Key Laboratory of Reproductive Biology, Institute of Zoology, Chinese Academy of Sciences, Beijing, China, 100101.

[4] Department of Animal Science, Iowa State University, Ames, IA USA

[5] ProGenetics, LLC, Blacksburg, VA, 24060, USA

[6] LFB USA, Inc.175 Crossing Blvd. Framingham, MA 01702

Correspondence and requests for materials should be addressed to R.S.P (Pratherr@missouri.edu) or to W. H. V. (wvelander2@unl.edu)

Supplementary Information contains:

Supplementary Figures S1-S9

Supplementary Tables S1-S9

Supplementary References

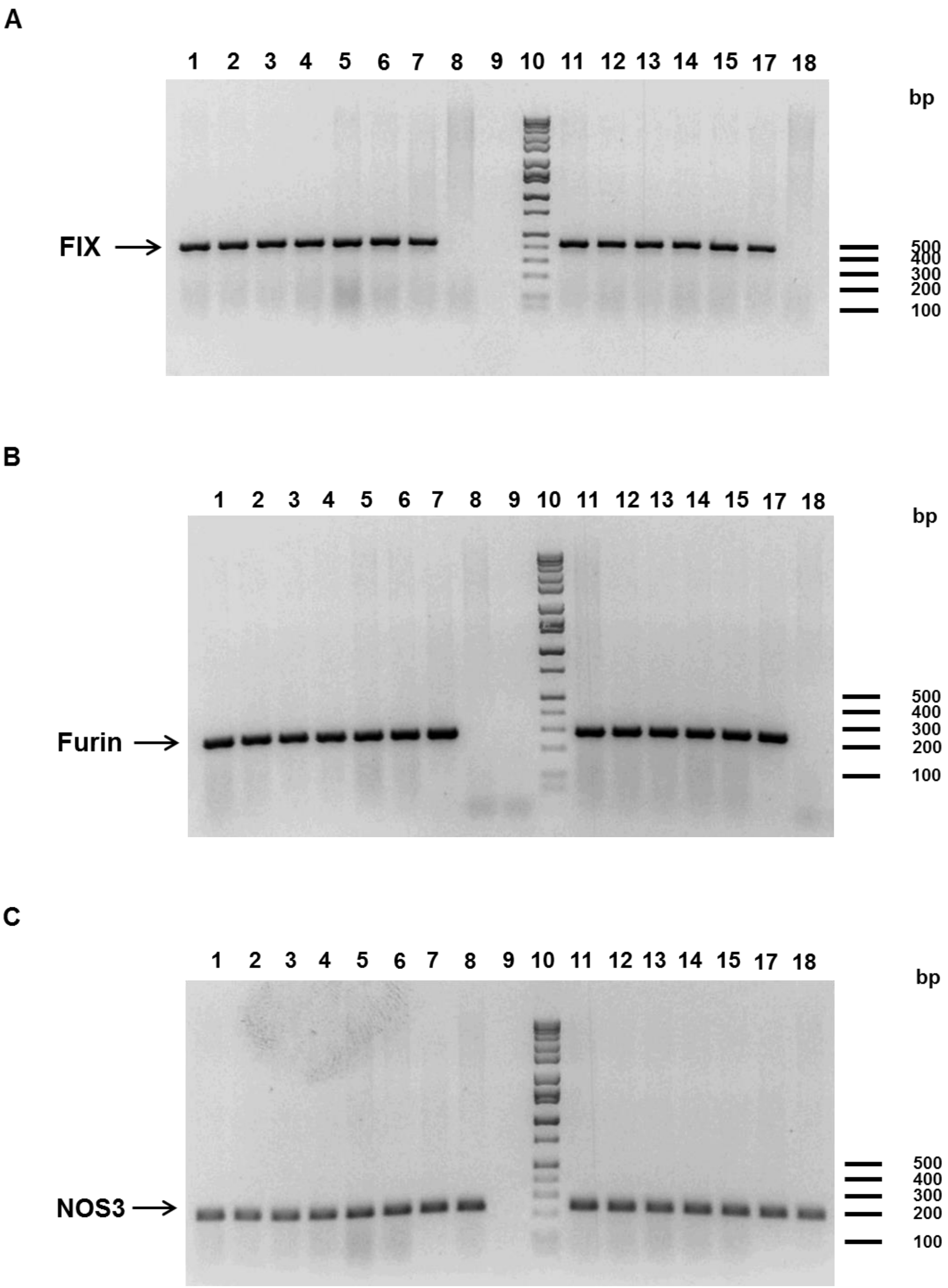
A
1 2 3 4 5 6 7 8 9 10 11 12 13 14 15 17 18
bp
FIX
500
400
300
200
100
B
1 2 3 4 5 6 7 8 9 10 11 12 13 14 15 17 18
bp
Furin
500
400
300
200
100
C
1 2 3 4 5 6 7 8 9 10 11 12 13 14 15 17 18
bp
NOS3
500
400
300
200
100

D

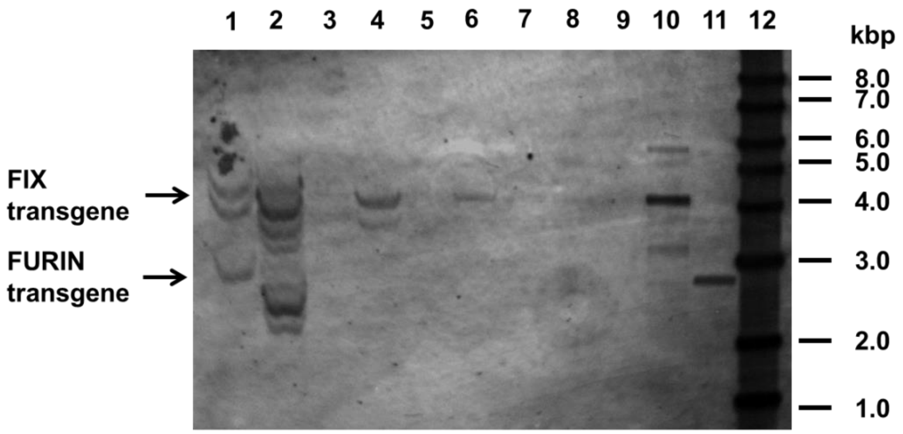

Supplementary Figures S1. Panel A. Genotyping of FIX and FURIN in the piglets of litter 78 and 79 by PCR. Eleven piglets from the two litters were bigenic for both transgenes; NOS3 was used as an endogenous gene positive control. Lane 1- 6, 6 piglets of litter 78; Lane 7, positive control; Lane 8, wild type genomic DNA; Lane 9, negative control; Lane 10, DNA reference ladder; Lane 11-15, 5 piglets of litter 79; Lane 16, positive control; Lane 17, wild type DNA; Lane 18, negative control. Panel D. Southern analysis of transgenic pig DNA using a WAP specific probe. 4.2 kbp fragment was WAP6FIX construct and 2.7 kbp fragment indicating the WAP5FURIN construct. Lane 1 and 2, Bigenic pig R185 and R175, respectively; Lane 4 and 6, monogenic pig K108 and K115, respectively; Lane 8, non-transgenic pig; Lane 3, 5, 7 and 9, blank; Lane 10, FIX transgene standard applied at 5 copies; Lane 11, FURIN transgene standard applied at 5 copies; Lane 12, Marker, biotinylated molecular weight markers. The arrows indicate FIX and FURIN transgenes, respectively.

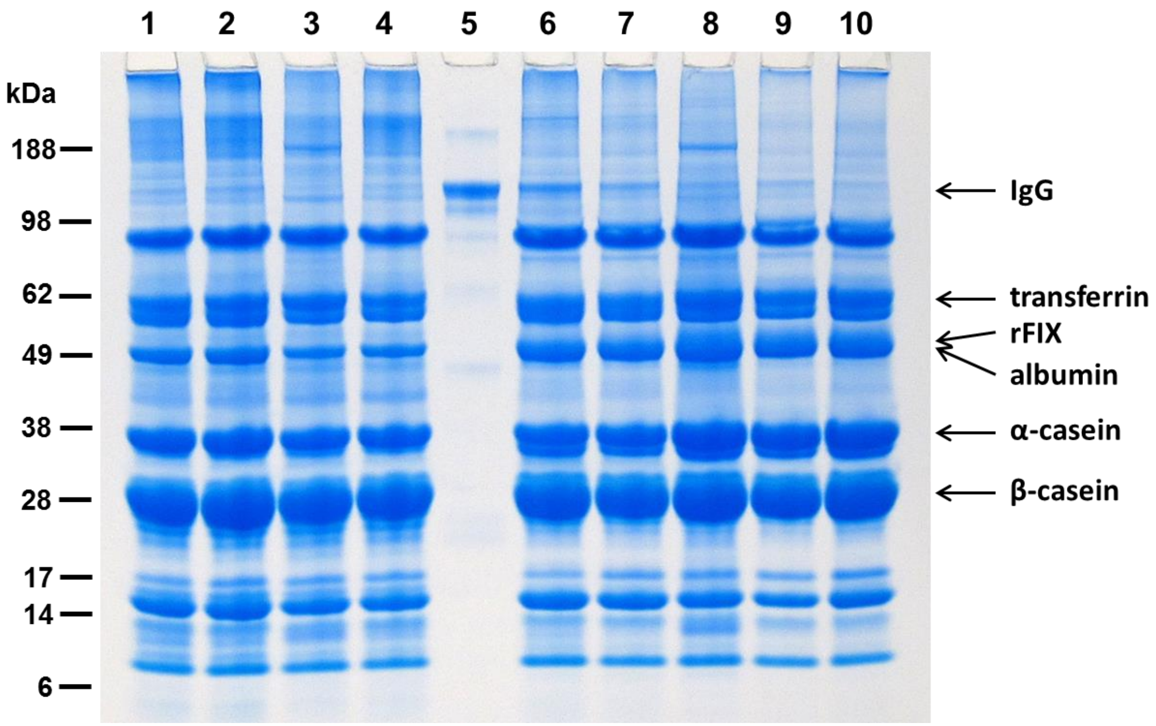

Supplementary Figures S2. Colloidal blue stained non-reduced SDS-PAGE analysis of endogenous milk protein composition of non-transgenic and bigenic pigs. Lane 1-4, milk samples from non-transgenic pig K90 (control) lactation day 12 (early stage), and 14 (middle stage), 30 and 35 (late stage), respectively, 2 µL skimmed milk sample each loaded; lane 5, pig serum IgG, 2 µg loaded; lane 6-10, milk samples from transgenic pig R180 lactation day 5 and 10 (early stage), 25 (middle stage), 31 and 35 (late stage), respectively, 2 µL skimmed milk sample each loaded.

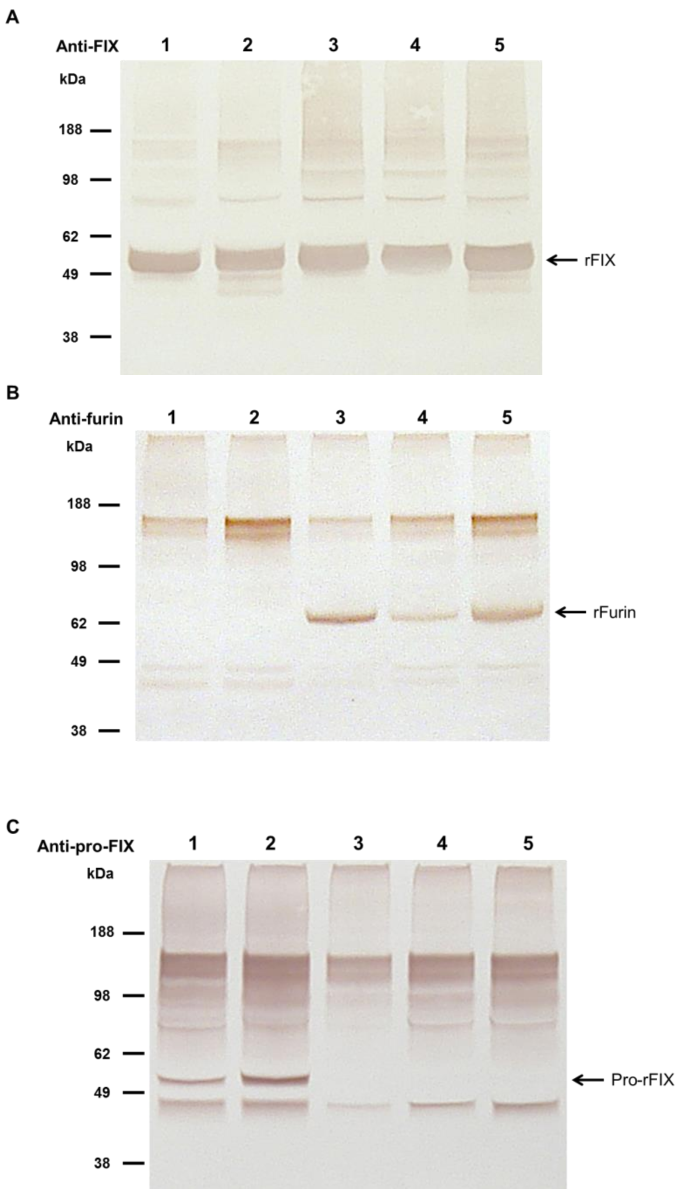

Supplementary Figure S3. Western blot analysis of milk samples from monogenic and bigenic pigs. Detection of A. rFIX antigen signal using anti-human FIX antibody; B. rFurin antigen using anti-human furin antibody; C. Pro-rFIX antigen using anti-human pro-FIX antibody. Milk sample pools from lactation days 5-35 of monogenic pigs (K75 and K101) and bigenic pigs (R175, R180 and R1014) were diluted with 200 mM EDTA 1: 1 and clarified as described in methods. Diluted milk sample pools: Lane 1, from pig K75; Lane 2, from pig K101; Lane 3, from pig R175; Lane 4, from pig R180; and lane 5,

from pig R1014. 2 μL of each diluted milk sample pool was loaded. The arrows indicate rFIX, rFurin and pro-rFIX signals.

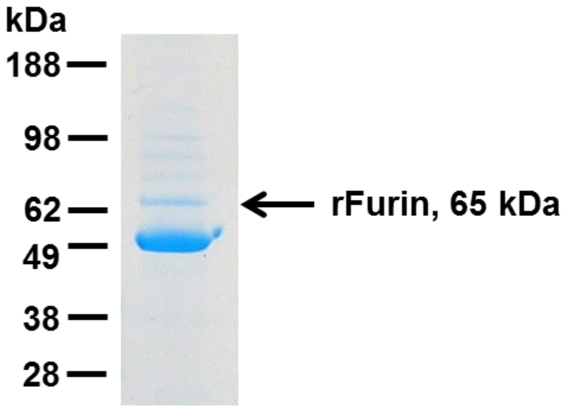

Supplementary Figure S4. Non-reduced Colloidal blue stained SDS-PAGE of partially purified rFurin. The 65kDa band indicated by arrow is the rFurin which exhibited an amino terminal sequence of mature human furin. (This is rFurin was partially purified from transgenic pig R185 and/or R1014 milk lactation pools). Four units of furin specific activity was applied and the amount of rFurin was estimated about 0.1 µg. The specific activity of this rFurin is about 4 x 10^4 U/mg. One unit of furin specific activity is defined as the cleavage of fluorogenic substrate pERTKR-AMC and producing 1 pmol AMC/min at room temperature.

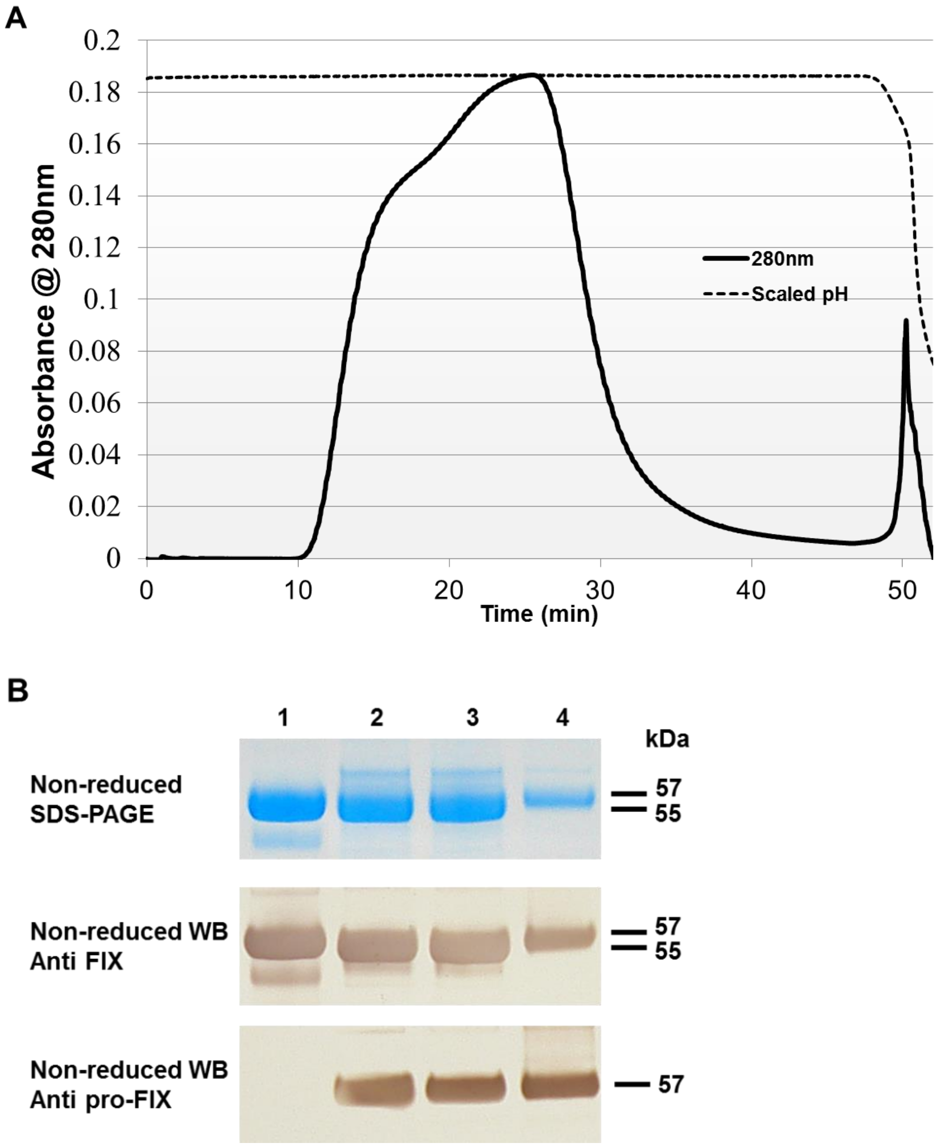

A
0.2
0.18
0.16
0.14
0.12
0.1
0.08
0.06
0.04
0.02
0
Absorbance @ 280nm
0
10
20
30
40
50
Time (min)
280nm
Scaled pH
B
1
2
3
4
kDa
Non-reduced SDS-PAGE
57
55
Non-reduced WB Anti FIX
57
55
Non-reduced WB Anti pro-FIX
57

C

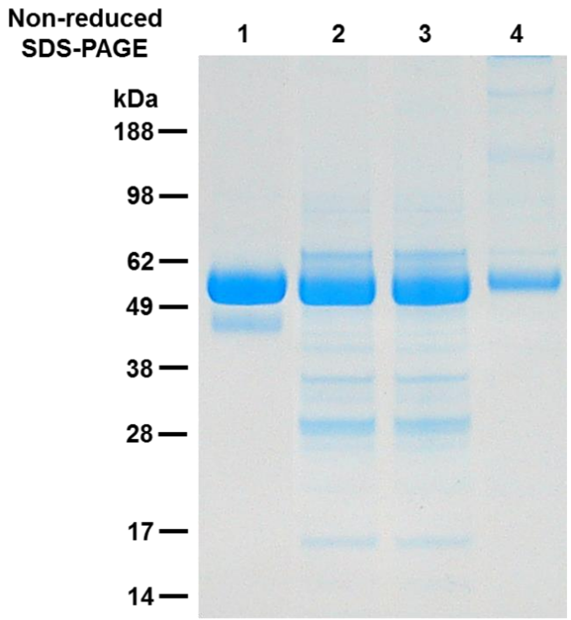
Non-reduced
SDS-PAGE
1
2
3
4
kDa
188
98
62
49
38
28
17
14

D

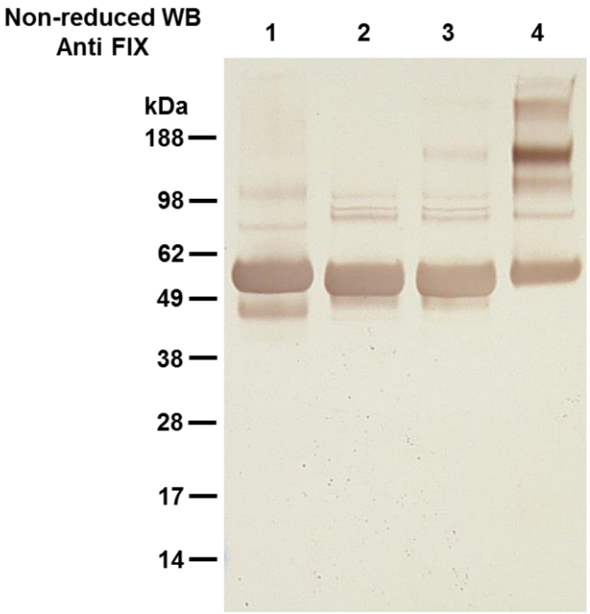
Non-reduced WB
Anti FIX
1
2
3
4
kDa
188
98
62
49
38
28
17
14

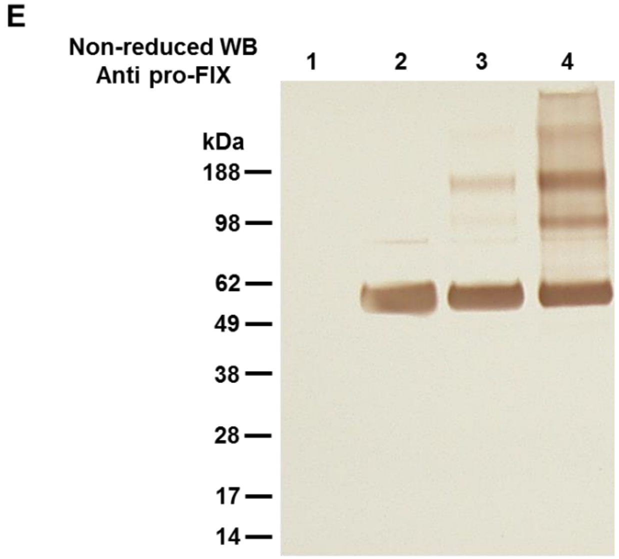

Supplementary Figure S5. Immunopurification of pro-rFIX from heparin sepharose processing of milk sample of monogenic pig. A. Chromatographic profile of pro-rFIX immunopurification. B. Non-reduced Colloidal blue stained SDS-PAGE and Western blot analysis using anti-FIX polyclonal antibody. Lane 1, pd-FIX, 6 µg loaded; Lane 2, loading sample from heparin sepharose eluate, 5 µg loaded; Lane 3, flow through of anti-pro-FIX immunoaffinity column, 5 µg loaded; Lane 4, elution from anti-pro-FIX immunoaffinity column, 5 µg loaded. This pro-rFIX product was used as a reference substrate for rFurin processing studies. Panel C, D and E are the parent images of non-reduced SDS-PAGE, non-reduced WB Anti FIX and non-reduced WB Anti pro-FIX in panel B, respectively.

A

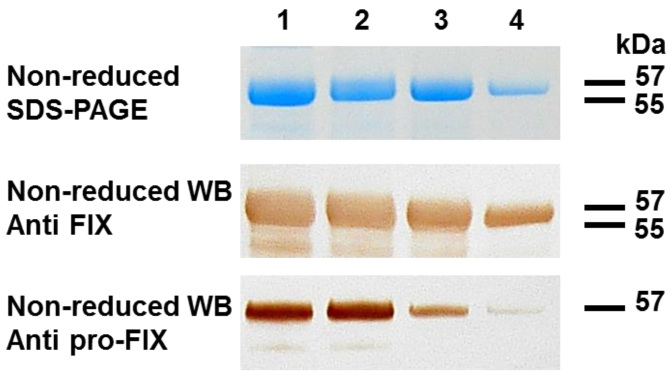
1
2
3
4
kDa
Non-reduced
SDS-PAGE
57
55
Non-reduced WB
Anti FIX
57
55
Non-reduced WB
Anti pro-FIX
57

B

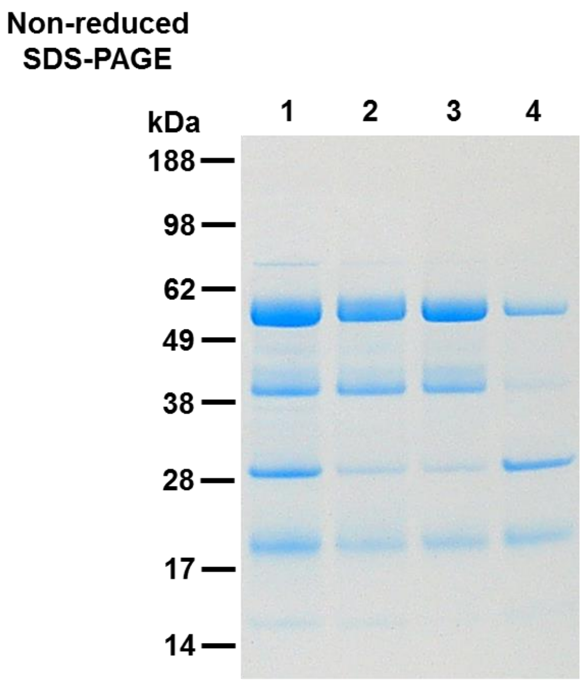
Non-reduced
SDS-PAGE
kDa
1
2
3
4
188
98
62
49
38
28
17
14

C

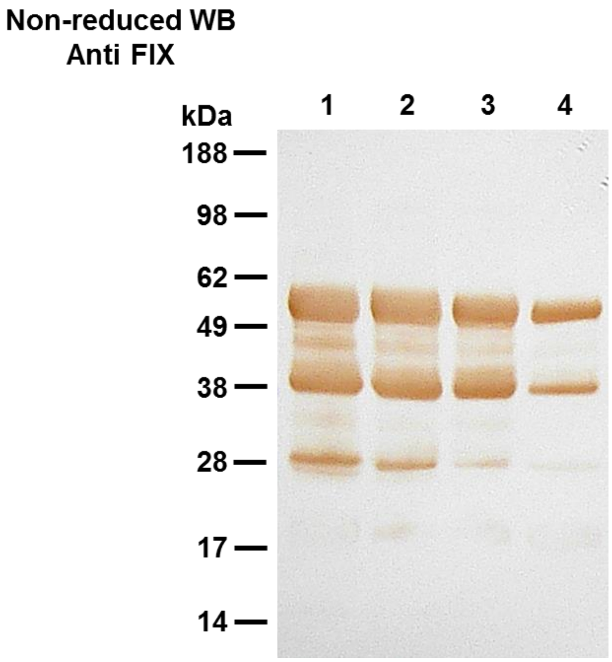

D

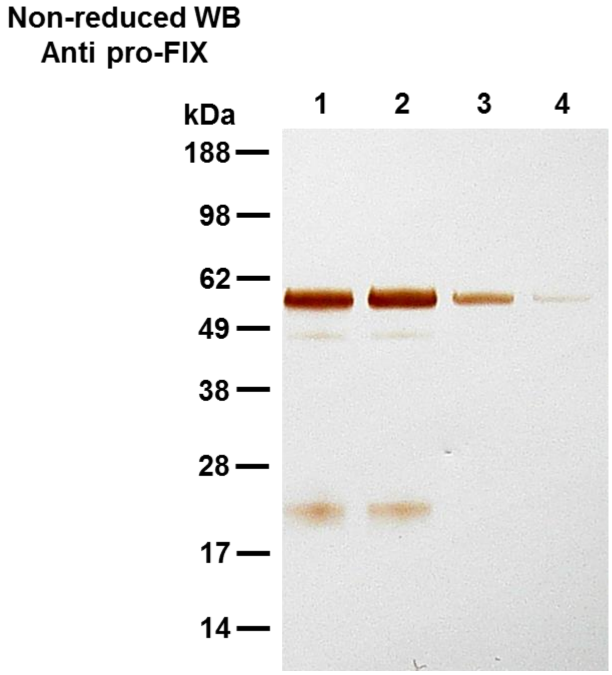

Supplementary Figure S6. Non-reduced Colloidal blue stained SDS-PAGE and Western blot analysis of high acidic rFIX and its HPLC-SEC fractions purified from monogenic milk. Panel A. Lane 1, rFIX high acidic fraction (loading sample of HPLC-SEC), 4 µg loaded; Lane 2, SEC pool 1, 2 µg

loaded; Lane 3, SEC pool 2, 2 µg loaded; Lane 4, SEC pool 3, 1 µg loaded. The concentration of each purified sample was measured by optical density with wavelength = 280 nm, $\varepsilon^{1\%}$ = 13.4. Note: milk samples were not treated *in vitro* by rFurin or bigenic milk before purification. Fractions of each SEC peak were pooled as in Fig 5; Panel B, C and D are the parent images of non-reduced SDS-PAGE, non-reduced WB Anti FIX and non-reduced WB Anti pro-FIX in panel A, respectively.

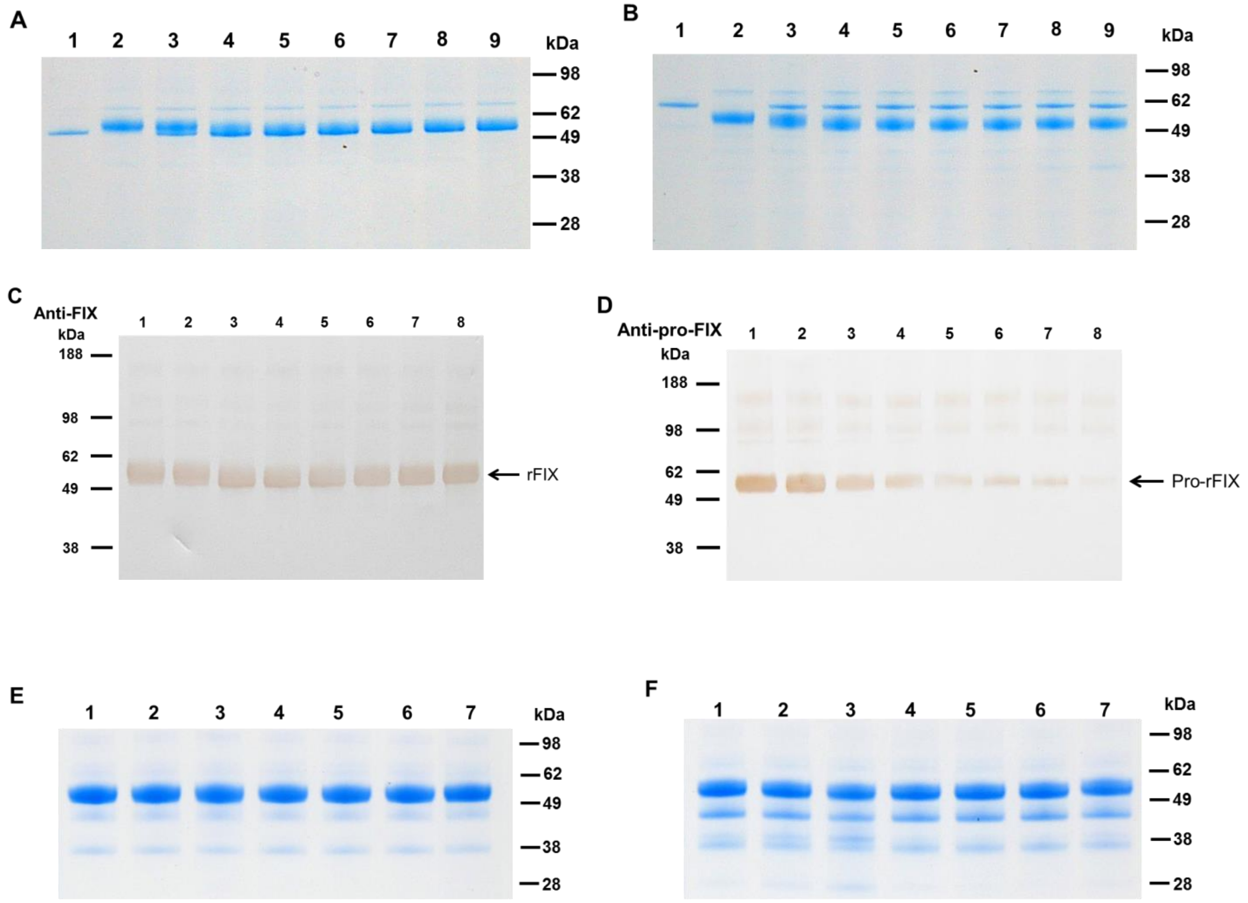

Supplementary Figures S7. Time course investigation of the processing of immunopurified pro-rFIX by partially purified rFurin. Panels A and B. Non- and reduced Colloidal blue stained SDS-PAGE gel respectively of pro-rFIX/rFurin reaction mass; Lane 1, partially purified rFurin reference, 0.075 U loaded; Lane 2, immunopurified pro-rFIX (57 kDa), 1 μg; Lane 3-9, samples of a mixture of partially purified rFurin (0.075 U) and immunopurified pro-rFIX (1 μg) at reaction times= 0, 30 min, 1, 2, 4, 8 and 24 hr, respectively. The arrows indicate rFIX and pro-rFIX signals, others are cross-reaction of antibody to the impurities in the samples. Panel C and D. Non-reduced Western blot analysis of total rFIX and pro-rFIX signal using anti-human FIX antibody and anti-human pro-FIX antibody, respectively; Lane 1, immunopurified pro-rFIX (57 kDa), 1 μg; Lane 2-8, samples of a mixture of partially purified rFurin (0.075 U) and immunopurified pro-rFIX (1 μg) at reaction times= 0, 30 min, 1, 2, 4, 8 and 24 hr, respectively. The arrows indicate rFIX and pro-rFIX signals, others are cross-reaction of antibody to the

impurities in the samples. Panels E and F. Non-reduced Colloidal blue stained SDS-PAGE of partially purified rFurin incubation with pd-FIX and purified functional rFIX. Lanes 1-7, reaction at T= 0, 30 min, 1, 2, 4, 8, 24 hrs, each lane contains reaction sample with initial content of 0.15 U rFurin and 2 μg pd- or rFIX.

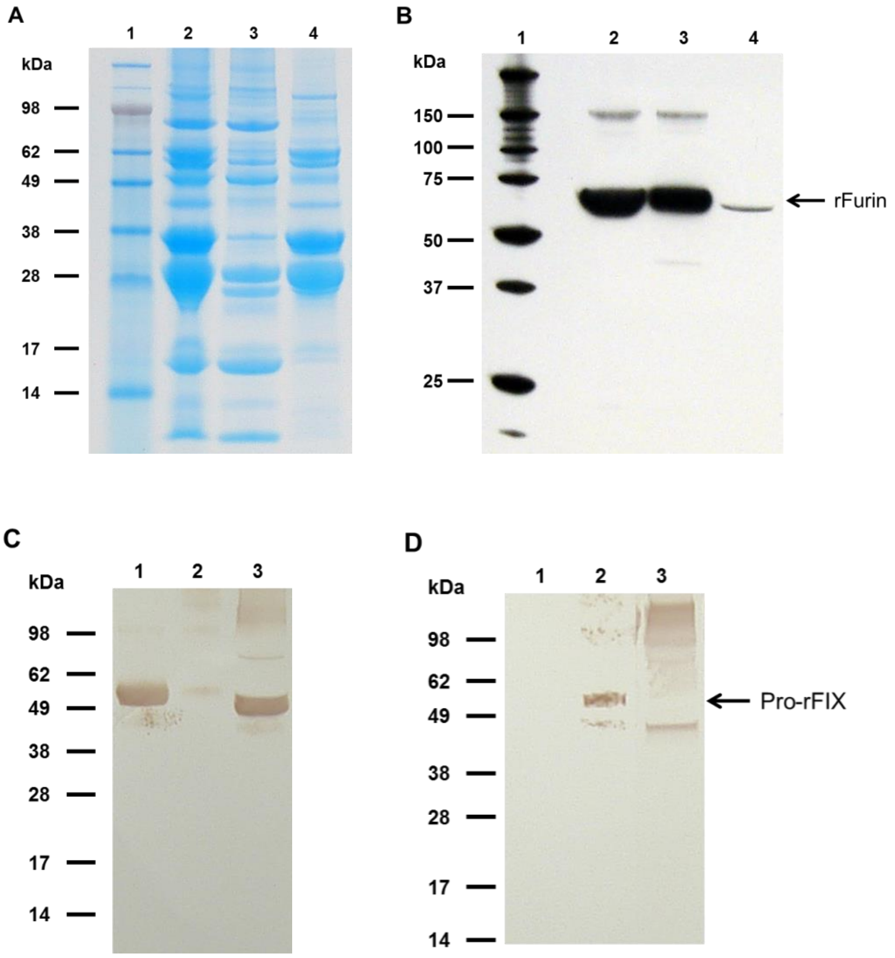

Supplementary Figure S8. Non-reduced SDS-PAGE and Western analysis of bigenic R185 milk sample for the presence of rFurin and pro-rFIX. Panel A is the colloidal blue stained non-reduced SDS-PAGE; and panel B is the non-reduced western blot developed by anti-human furin antibody. Lane 1. molecular ladder; lane 2. R185 whole milk, 2 µL loaded; lane 3. R185 skimmed milk, equivalent 2 µL whole milk loaded; lane 4, R185 casein pellet, equivalent 2 µL whole milk loaded; lane 5. The arrows indicate rFurin signals. Non-reduced Western analysis of bigenic R185 milk sample; panel C. anti-human FIX; and panel D. anti-human pro-FIX; Lane 1. pd-FIX reference, 4 µg loaded; lane 2. immuno-purified pro-rFIX reference, 0.2 µg loaded; lane 3. R185 milk lactation day pool, respectively, 2 µL whole milk loaded. The arrow indicates pro-rFIX signals.

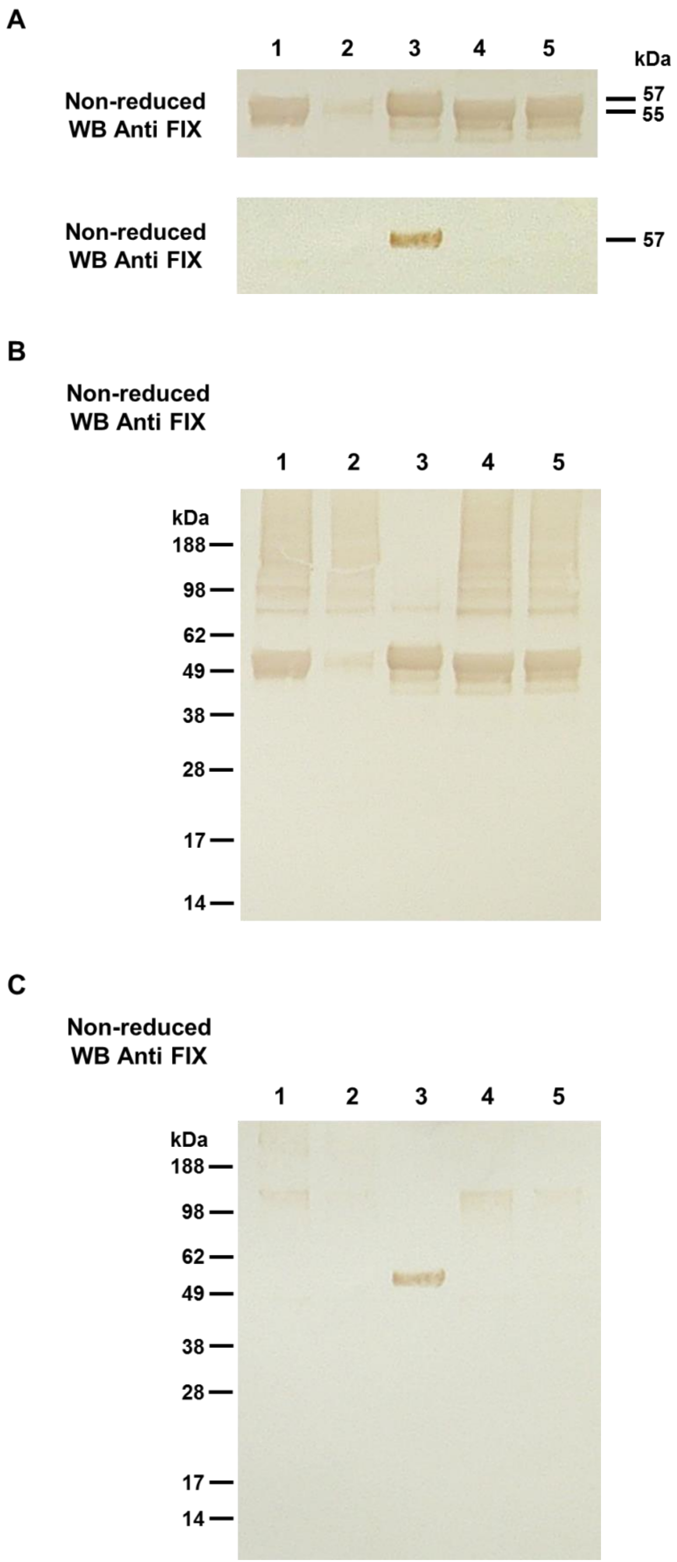
A
1 2 3 4 5
kDa
Non-reduced
WB Anti FIX
57
55
Non-reduced
WB Anti FIX
57
B
Non-reduced
WB Anti FIX
1 2 3 4 5
kDa
188
98
62
49
38
28
17
14
C
Non-reduced
WB Anti FIX
1 2 3 4 5
kDa
188
98
62
49
38
28
17
14

Supplementary Figure S9. Western blot analysis of pro-rFIX to rFIX processing by rFurin. Panel A. Lane 1, bigenic pig R175 milk sample, 0.8 µL loaded; Lane 2, rFIX stripped bigenic pig R175 milk sample, 0.8 µL loaded; Lane 3, monogenic pig K96 milk sample, 0.8 µL loaded; Lane 4, monogenic pig K96 milk sample incubated with rFIX stripped bigenic pig R175 milk sample at 2:1 (v/v) ratio at 4℃ for 1.5 hour, 1.2 µL loaded; Lane 5, monogenic pig K96 milk sample incubated with rFIX stripped bigenic pig R175 milk sample at 4:1 (v/v) ratio at 4℃ for 1.5 hour, 1 µL loaded. Note. All samples were whole milk without EDTA or protease inhibitor treatment; panel B and C are the parent images for non-reduced WB anti FIX and pro-FIX in panel A, respectively.

Supplementary Table S1. rFIX biosynthesis in Pig Mammary Epithelia (PME) and Baby Hamster Kidney (BHK) Cells.

Host	PME cell*		BHK cell**
Cell Density (cells/mL)	10^9		10^6
Total rFIX expression level (pg/cell/hour)	0.13[†]	1.3[††]	0.67
% Functional rFIX of total rFIX	100%	10%	18%
Functional rFIX expression level (pg/cell/hour)	0.13	0.13	0.12
Total rFIX bioreactor output rate (µg/mL/hour)	200	2000	0.67
Functional rFIX bioreactor output rate (µg/mL/hour)	200	200	0.12

* The average total volume of the each individual pig mammary gland during the lactation is about 250 mL with 30-40% gland volume being secretory PME cells. The PME cell density of the gland is about 10^9 cell/mL secretory duct volume[1]. The rFIX throughput in pig milk was quantified assuming an hourly pig milk letdown. Total 8-10 functional glands with each one letdowns about 50-75 mL milk per hour. Milk can be harvested from each pig for commercial purposes about 3 times per day with the remaining milk letdown dedicated to piglets. A pig lactation is sustainable for about 40 days in the presence of piglets with 2 lactations resulting from two pregnancies with farrowing per year.

** Wajih et. al. reported on the production of recombinant human FIX in BHK cells at about 16 µg total FIX protein/day/10^6 cells with 18% consisting of functional rFIX[2]. The rFIX concentration was monitored and allowed to accumulate in the BHK cell culture every 24 hours.

[†] Using a short (2.5 kbp) WAP mammary specific promoter, we observed an overall expression level of rFIX in the milk of transgenic pigs about 200 µg/mL or less. In this case, all rFIX was highly carboxylated (10, 11 and 12 Glas) and possessed a native or better pro-coagulant activity and an amino terminal, amino acid sequence[3].

†† Using the long (4.1 kbp) WAP mammary specific promoter, we observed an overall expression level of rFIX in the milk of transgenic pigs of about 2,000 μg/mL. However, only about 10% or less of rFIX was highly carboxylated (10, 11 and 12 Glas) and possessed a pro-coagulant activity and native, amino-terminal amino acid sequence[4].

Supplementary Table S2. Summary of SCNT production of bigenic founder pigs containing long WAP-FIX and short WAP-FURIN.

A.

Donor cells	embryos transferred *	Numbers of Piglets born	Numbers of Bigenic pig	% Bigenic Founder (F0)
male	152	NP**	NP**	100%
	213	6	6	
	162	5	5	
	180	NP**	NP**	
	182	4	4	
	214	NP**	NP**	

* Embryos transferred per surrogate recipient

** No pregnancy.

For male production, 6 embryo transfers were performed and 3 surrogates were developed to term. Totally 15 cloned piglets were born and all of them are transgenic.

B.

Founder (F0) (Male)	F1 Animal (Female)	Total Offspring	Bigenic	Non-Transgenic	Percentage
80-4	R175	8*	4	4	50%
78-2	R180	11*	6	5	55%
79-2	R185	14**	8	7	57%
79-2	R1014	6*	6	0	100%

* Transgenic pig R175, R180 and R1014 each had single litter.

** Transgenic pig R185 had 2 litters with 6 of 7 and 2 of 7 being bigenic, respectively.

Supplementary Table S3. N-terminal amino acid (a.a.) sequence of detected rFurin, pro-rFIX, and rFIX in milk samples from transgenic pigs

Molecular Weight (kDa)*	N-terminal a.a. Sequence	Detected Protein**
65	Asp-Val-Tyr-Gln-Glu	rFurin
57	Thr-Val-Phe-Leu-Asp	pro-rFIX
55	Tyr-Asn-Ser-Gly-Lys	rFIX

* The molecular weight of the protein samples is in the non-reduced condition.

** Protein samples detected in the transgenic milk samples using Western blot developed by specific antibodies (described in methods). The protein samples were purified and analyzed their N-terminal amino acids sequence. rFurin is only detected in the bigenic milk samples; Pro-rFIX is only detected in the monogenic milk samples; and the rFIX is detected in both monogenic and bigenic milk samples.

Supplementary Table S4. Formulation of rFurin using various surfactants to stabilize PACE activity.

**rFurin Activity before chromatography* %**	100
**rFurin Activity after chromatography without surfactant %**	44
**rFurin Activity after chromatography with (0.1% Brij-35) %**	92
**rFurin Activity after chromatography with (0.4 mM DDM) %**	86

* rFurin was purified by using the ceramic hydroxyapatite (CHT) chromatography. The percent specific activity of chromatographic fractions containing rFurin was assayed as in methods.

Supplementary Table S5. Purification table of rFurin from milk samples from bigenic pig R1014

Purification Steps	Volume (mL)	Activity (U/mL)	Total Activity (U)	Activity Recovery %
**Whole Milk**	10	1925	19247	100
**Clarified Milk**	45	323	14527	75
**DEAE**	85	142	12040	63
**CHT**	175	50	8666	45
**Q**	135	53	7209	37

Supplementary Table S6. Time course study of *in vitro* pro-rFIX to rFIX processing by partially purified rFurin.

Sample	Pro-rFIX (µg)*	Pro-rFIX %**	Total rFIX (µg)*	Total rFIX %**	Activity (IU/mg) ††
pro-FIX	N/A				1.2 ± 0.2
Rxn t=30 min	0.38 ± 0.05	63	0.66 ± 0.05	88	14.1 ±1.3
Rxn t= 1 hour	0.40 ± 0.05	67	0.72 ± 0.03	96	23.7 ± 2.4
Rxn t= 2 hour	0.15 ± 0.03	20	0.67 ± 0.00	89	30.1 ± 2.0
Rxn t= 4 hour	0.00 ± 0.02†	10†	0.66 ± 0.14	89	29.0 ± 8.5
Rxn t= 8 hour	-0.11 ± 0.01†	4†	0.72 ± 0.04	96	32.1 ± 3.8
Rxn t= 24 hour	N/A				35.4 ± 4.1
Rxn t= 24 hour (Control)	0.56 ± 0.03	94	0.83 ± 0.04	110	3.5 ± 0.7

* The amount of each pro-rFIX and total rFIX of rFurin processing pro-rFIX sample was calculated from quantitative Western blots, the value is mean ± standard deviation (n=3)

** The amount of remaining pro-rFIX respective total rFIX in each reaction sample was achieved by using the amount of pro-rFIX and total rFIX loaded on the blot and the volume loaded compared to the initial amount before the reaction.

† Both reaction samples (4 and 8 hours) contain very little pro-rFIX amount to be detected (less than the detecting range on Western blot analysis). The percentage of the remaining pro-rFIX were estimated (using the lowest detecting range).

†† The specific activity values are mean ± standard deviation.

Supplementary Table S7. Analysis of healthy phenotypic characteristic of monogenic FIX versus bigenic FIX/furin pigs.

Pig ID	Genotype	Litter Size	Length of Lactation	Milk vol./teat/milking (mL)
**K96**	Monogenic	9	37 days (1st Lactation)	61
**K94**	Monogenic	6	45 days (2nd Lactation)	74
**K89**	Monogenic	11	45 days (2nd Lactation)	41
**R185**	Bigenic	7	40 days (1st Lactation)	50
**R185**	Bigenic	7	47 days (2nd Lactation)	55

Supplementary Table S8. Estimated impact of PTM bioengineering on capacity to meet clinical demand in developing countries.

Mammary specific promoter	Short promoter		Long promoter	
ER-Golgi bioengineering*	No	Yes	No	Yes
rFIX protein concentration (µg/mL)	200		2000	
Rate of functional rFIX biosynthesis (pg/cell/hour)	0.13	0.13	0.13	0.34
Functional rFIX concentration (µg/mL)	200	200	200	555
Annual units production of functional rFIX (IU/pig)**	1.6×10^6	1.6×10^6	1.6×10^6	4.4×10^6
Number of pigs needed to meet clinical demand in developing countries†	2500	2500	2500	900

* Wajih et. al. reported producing recombinant human FIX in BHK cells at about 16 µg /day/10^6 cells and engineered the cell to overexpress VKORC1 (an 18-kDa subunit of VKOR) and increase the functional rFIX from 18% to 50%, which is about 2.8-fold[2]. We assumed that bioengineering both ER and Golgi by coexpression of rVKOR and rFurin with rFIX in pig mammary gland could increase the expression level of functional rFIX by 2.8-fold.

** The milk generated by transgenic pigs for commercial function is about 200 L/year/pig. Assuming the yield of the functional rFIX is about 40% of the original 10% in the milk. Considering about 50% of the functional rFIX is degraded or activated by protease in the milk, the overall yield of functional rFIX from the milk is about 2%.

† Estimated 4 billion IU rFIX required for meeting the clinical demand in developing countries.

Supplementary Table S9. PCR oligonculeotide primers and product fragment size used for identification of FIX/FURIN transgenes in transgenic pigs.

Gene	Primers	Amplicon Size
**F9**	Forward: 5'-TACCTCTTTGGCCGATTCAG-3'	430 bp
	Reverse: 5'-GCTCCTTCATTTTCTCCGCT-3',	
**Furin:**	Forward: 5'- GCGAGAGGACCGCCTTTATCAAAGA-3'	293 bp
	Reverse: 5'-TGGAAATCACCTGTGGCTGTCTTGC-3'	
**NOS3**	Forward: 5' -ACGAGCCTCCAGAACTCTTTGCTT-3'	243 bp
	Reverse: 5'- TTTCCAGCAGCATGTTGGACACTG-3′	

Reference

1. Morcol, T. *et al.* The porcine mammary gland as a bioreactor for complex proteins. *Ann N Y Acad Sci* **721**, 218-33 (1994).
2. Wajih, N., Hutson, S.M., Owen, J. & Wallin, R. Increased production of functional recombinant human clotting factor IX by baby hamster kidney cells engineered to overexpress VKORC1, the vitamin K 2,3-epoxide-reducing enzyme of the vitamin K cycle. *J Biol Chem* **280**, 31603-7 (2005).
3. Van Cott, K.E. *et al.* Transgenic pigs as bioreactors: a comparison of gamma-carboxylation of glutamic acid in recombinant human protein C and factor IX by the mammary gland. *Genet Anal* **15**, 155-60 (1999).
4. Lindsay, M. *et al.* Purification of recombinant DNA-derived factor IX produced in transgenic pig milk and fractionation of active and inactive subpopulations. *J Chromatogr A* **1026**, 149-57 (2004).
